# Semi-Synthesis, Cytotoxic Evaluation, and Structure—Activity Relationships of Brefeldin A Derivatives with Antileukemia Activity

**DOI:** 10.3390/md20010026

**Published:** 2021-12-24

**Authors:** Xu-Xiu Lu, Yao-Yao Jiang, Yan-Wei Wu, Guang-Ying Chen, Chang-Lun Shao, Yu-Cheng Gu, Ming Liu, Mei-Yan Wei

**Affiliations:** 1 Key Laboratory of Marine Drugs, The Ministry of Education of China, School of Medicine and Pharmacy, Ocean University of China, Qingdao 266003, China; xuxiulu@126.com (X.-X.L.); jiangyaoyao1226@163.com (Y.-Y.J.); wuyanwei1214@163.com (Y.-W.W.); shaochanglun@163.com (C.-L.S.); 2 Laboratory for Marine Drugs and Bioproducts, Qingdao National Laboratory for Marine Science and Technology, Qingdao 266200, China; 3 Key Laboratory of Tropical Medicinal Resource Chemistry of Ministry of Education, College of Chemistry and Chemical Engineering, Hainan Normal University, Haikou 571158, China; chgying123@163.com; 4 Syngenta Jealott’s Hill International Research Centre, Bracknell RG42 6EY, UK; yucheng.gu@syngenta.com; 5 State Key Laboratory for Chemistry and Molecular Engineering of Medicinal Resources, Guangxi Normal University, Guilin 541001, China; 6 College of Food Science and Engineering, Ocean University of China, Qingdao 266003, China

**Keywords:** brefeldin A, ester derivative, chronic myelogenous leukemia, BCR-ABL, proliferation inhibition, molecular modeling

## Abstract

Brefeldin A (**1**), a potent cytotoxic natural macrolactone, was produced by the marine fungus *Penicillium* sp. (HS-N-29) from the medicinal mangrove *Acanthus ilicifolius*. Series of its ester derivatives **2**–**16** were designed and semi-synthesized, and their structures were characterized by spectroscopic methods. Their cytotoxic activities were evaluated against human chronic myelogenous leukemia K562 cell line in vitro, and the preliminary structure–activity relationships revealed that the hydroxy group played an important role. Moreover, the monoester derivatives exhibited stronger cytotoxic activity than the diester derivatives. Among them, brefeldin A 7-*O*-2-chloro-4,5-difluorobenzoate (**7**) exhibited the strongest inhibitory effect on the proliferation of K562 cells with an IC_50_ value of 0.84 µM. Further evaluations indicated that **7** induced cell cycle arrest, stimulated cell apoptosis, inhibited phosphorylation of BCR-ABL, and thereby inactivated its downstream AKT signaling pathway. The expression of downstream signaling molecules in the AKT pathway, including mTOR and p70S6K, was also attenuated after **7**-treatment in a dose-dependent manner. Furthermore, molecular modeling of **7** docked into **1** binding site of an ARF1–GDP-GEF complex represented well-tolerance. Taken together, **7** had the potential to be served as an effective antileukemia agent or lead compound for further exploration.

## 1. Introduction

Chronic myelogenous leukemia (CML) is characterized by the translocation of chromosomes 9 and 22, which generates the BCR-ABL fusion oncogene with constitutively active tyrosine kinase [1]. This aberrant tyrosine kinase exerts its oncogenic function for malignant transformation mainly by activating multiple cellular signaling pathways, including the PI3K/AKT, MAPK/ERK, and JAK-STAT, which also contributes to the insensitivity of chemotherapy drugs [2,3]. Imatinib mesylate (Gleevec) was selected as the first tyrosine kinase inhibitor (TKI) that has been proven to be an effective agent in CML treatment. However, the emergence of drug resistance is becoming a common problem for the failure of imatinib treatment [4]. The second-generation TKIs, such as nilotinib, dasatinib, and bosutinib, were then employed to overcome acquired resistance to imatinib [5,6,7]. Though they show a significant effect on imatinib-resistant patients, the effect on those patients carrying T315I mutation is limited [8]. Therefore, novel agents to improve therapeutic outcomes of CML are needed urgently.

**Figure 1:**
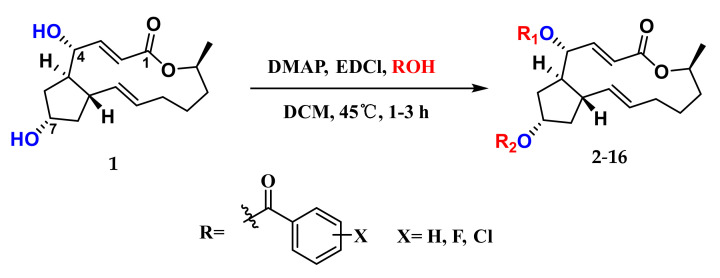
Synthesis of the derivatives **2**–**16** of brefeldin A (**1**).

Marine secondary metabolites with novel structures and wide-ranging biological activities have been proven to be rich sources of chemical entities for drug discovery [9,10]. Brefeldin A (BFA, **1**, Figure 1), a 13-membered macrolactone with a cyclopentane substituent, was first isolated from *Penicillium decumbens* in 1958 [11] and subsequently identified as a metabolite from the marine-derived fungus *Penicillium* sp. PSU-F44 [12], *Penicillium janthinellum* DT-F29 [13,14], as well as *Penicillium* sp. (CGMCC No.17193) recently published by our group [15], which is the same as marine fungus *Penicillium* sp. (HS-N-29) described here. BFA (**1**) impairs the small G-protein ARF1 (ADP ribosylation factor 1) activation by hindering its association with its large guanine nucleotide exchange factor (GEF) enzyme [16,17], which induces the breakdown of vesicle-mediated protein transport, thus, causes the Golgi complex redistributing into the endoplasmic reticulum (ER) [18,19,20]. Structural insights into the uncompetitive inhibitory mechanism of **1** conduct to an abortive pentameric ARF1–Mg^2+^–GDP–BFA–Sec7 complex, and **1** was described as an interfacial inhibitor [21]. Previous studies reported that **1** showed obvious anticancer activity in a variety of cancers, including colorectal, prostate, lung, and breast cancers [22,23], which has been touted as a promising lead molecule for anticancer drug development. However, **1** showed some undesirable limitations due to its low bioavailability, high toxicity, and poor pharmacokinetics [24,25]. Therefore, it is of great necessity to synthesize the derivatives of **1** with kept or enhanced potency and decreased toxicity simultaneously. In fact, a number of derivatives of **1** have been reported in conjunction with anticancer investigations [25,26,27,28,29,30,31]. The existing information on structure–activity relationships (SARs) of **1** indicated the significant influences of *α*, *β*-unsaturated lactone, alkenes, and conformational rigidity of the molecule on its cytotoxicity [26,30]. Most of the derivatives generated in the way that alters the above moiety of the structure showed no or reduced biological activity compared with **1** [27,28]. However, sulfide and sulfoxide prodrugs of **1** showed promising antitumor effects both in vitro and in vivo due to its yield of an intact pharmacophore of **1** after drug metabolism [29]. Moreover, C15-substituted analogs were also reported to exhibit significant activities tested at the NCI [31]. Previous studies outlined the ester derivatives at 4-OH or 7-OH group of **1** generally displayed excellent cytotoxicity against different cancer cell lines [23,25]. However, the lack of a clear understanding of **1** and its derivatives in CML cells inhibition limits its usefulness as a lead compound for antileukemia agents. The detailed mechanism supporting the antileukemia effect of **1** and its derivatives needs to be further elucidated. That provides a rational and effective strategy for structure modification and mechanism study.

In the present study, the crude extracts of 55 marine fungal strains from the medicinal mangrove *Acanthus ilicifolius* were evaluated, and the potent natural compound **1** was discovered in the extract of the *Penicillium* sp. (HS-N-29) strain under the guidance of cytotoxic activity. Our focus was to design and synthesize a series of new derivatives (**2**–**16**) of **1**, and evaluate their cytotoxicities against K562 cells. Of them, compound 7, the most active one, arrested cell cycle at G0/G1 phase, stimulated caspase-dependent cell apoptosis, inhibited phosphorylation of BCR-ABL, and thereby inactivated its downstream AKT signaling pathway. The present results provided evidence that **7** exhibited the great potential to be developed as an antileukemia agent.

## 2. Results and Discussion

### 2.1. Chemistry

The excellent cytotoxic activity, along with poor pharmacokinetic properties of **1**, prompted us to semi-synthesize a series of derivatives to explore the potency of this class of molecules. Natural **1** was obtained by fermentation of the *Penicillium* sp. (HS-N-29) strain. Detailly, the fungal strain HS-N-29 was cultivated in a PDB medium (200.0 g of potato, 20.0 g of glucose in 1 L of seawater) at 28 °C with shaking for 2 weeks, and subsequently extracted with EtOAc. The EtOAc extract was subjected to silica gel and then recrystallized to produce compound 1. The structure of 1 was confirmed by NMR data analysis compared with the literature [30] and single-crystal X-ray diffraction analysis.

**Figure 2:**
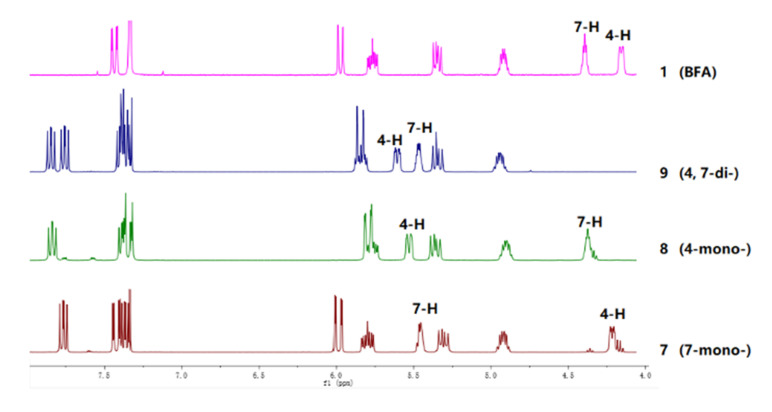
The partial stacking ^1^H NMR spectra of **1** and its derivatives **7**–**9**.

Given that both the aryl esters and C15-aryl-substituted analogs of **1** displayed efficient cytotoxicities [25,31], as well as halogen substitution played an important role in the regulation of diverse bioactivities of compounds [32,33], the semi-synthesis of various halogenated benzoic acids of **1** was carried out and outlined in Figure 1. Simply, compound **1** reacted with different halogen-substituted benzoic acids in the presence of 4-dimethylaminopyridine (DMAP, catalyst) and 1-ethyl-3-(3-dimethylaminopropyl) carbodiimide hydrochloride (EDCl, dehydrating agent) afforded thirteen new ester derivatives **4**–**16** and two known derivatives **2**–**3**. All of them were separated by using repeated reversed-phase silica column chromatography combined with semi-preparative HPLC. The structures of new compounds were characterized by ^1^H NMR, ^13^C NMR, and HRESIMS. It is worth noting that the position of esterification at 4-OH or 7-OH was the key point to structural identification, which was distinguished by the changes of their corresponding chemical shift (*δ*) values of H-4 or H-7. It followed such a regular pattern that the *δ* value of H-4 or H-7 shifted by nearly 1–1.5 ppm downfield after esterification. Three corresponding derivatives **7**, **8**, and **9** of 2-chloro-4,5-difluorobenzoic acid were used to illustrate the detailed structural determination (Figure 2). In detail, comparisons of their partial ^1^H NMR spectra with that of **1** in the interval of *δ*_H_ 4.0–8.0 ppm, where the key proton signals appeared, the chemical shift of H-7 in 7-monoester derivative **7** shifted from 4.34 to 5.38 ppm with a slight deviation on the chemical shift of H-4 (*δ*_H_ 4.16 in **7**, *δ*_H_ 4.11 in **1**). Meanwhile, the chemical shift of H-4 in 4-monoester derivative **8** shifted from 4.11 to 5.48 ppm with no difference in the chemical shift of H-7 (*δ*_H_ 4.33 in **8**, *δ*_H_ 4.33 in **1**). The chemical shifts of H-4 and H-7 in 4,7-diester derivative **9** shifted from 4.11 to 5.56 ppm and 4.34 to 5.41 ppm, respectively. On this basis, the structures of all the derivatives were confirmed.

### 2.2. Biological Evaluation

#### 2.2.1. Cytotoxic Activity

The cytotoxicity of the natural product **1** together with its derivatives **2**–**16** were evaluated against human chronic myelogenous leukemia cell line K562 with doxorubicin as a positive control. The results showed that the mono-substituted derivatives displayed moderate to strong cytotoxicity against K562 cells with IC_50_ values ranging from 0.84 to 2.49 µM (Table 1). Strikingly, the inhibitory effects of **4**, **5**, and **7** were superior to that of other compounds (**2**, **6**, **8**, **10**, **11**, **13**, and **14**) with the IC_50_ values of 0.91, 0.91, and 0.84 µM, respectively. Generally, the activity of 4-mono- or 7-monoester derivatives evidenced no obvious distinction. Decreased cytotoxicity was observed on derivatives **3**, **9**, **12**, and **15** with di-substitution of both -OH groups at positions C4 and C7, indicating that the -OH groups played an important role in cytotoxic property. Moreover, in comparison to **2**, a stronger activity was observed on **5**, **7**, **10**, and **13**, which indicated that the introduction of fluorine or chlorine atoms contributed to the cytotoxicity to some extent, but it was not affected by the number and position of halogen atoms. Significantly, derivative **7** with the structure featuring a 2-chloro-4,5-difluoro-substituted benzene ring greatly suppressed K562 cells growth with an IC_50_ value of 0.84 µM, which was better than that of other compounds and slightly lower than that of **1** (IC_50_ ratio **7**/**1** = 3.5, in the same order of magnitude). Given that the aqueous solubility of the compound may be more important to be considered in terms of drug testing, as well as drug administration, the solubility measurement of **7** was also indicated. Strikingly, **7** possessed improved water solubility (220 μg/mL), which was more than 3-fold higher than that of **1** (72 μg/mL). Therefore, it represented a potential promising lead compound and encouraged us to further investigate the possible cellular mechanisms of **7**.

#### 2.2.2. Compound **7** Inhibited the Proliferation of K562 Cells

**Figure 3:**
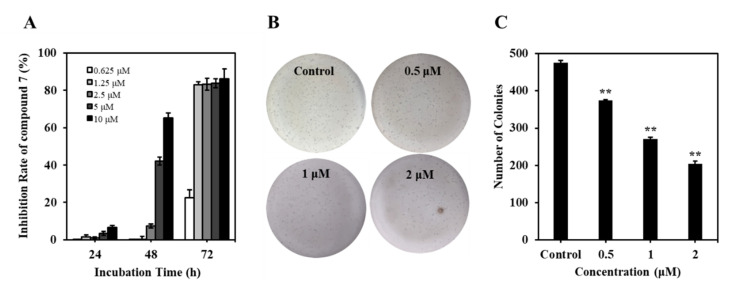
Compound **7** inhibited the proliferation of K562 cells. (**A**) Proliferation inhibition rates (%) of **7** against K562 cells. K562 cells were treated with **7** (0–10 μM) for 24–72 h. Cell viability was subjected to MTT assay. (**B**) Effect of **7** on soft agar colony formation of K562 cells. K562 cells were treated with 0–2 μM of **7** for indicated time. Cell proliferation was determined by colony formation. (**C**) Quantification of the colonies in soft agar. Values are expressed as mean ± SD of three independent experiments. ** *p* < 0.01, versus control.

To elucidate the inhibitory efficacy of **7**, the cell viability was detected using an MTT assay. Cultured K562 cells were treated with **7** for 24, 48, and 72 h, and the data showed that the **7**-treated group markedly decreased the cell viability in a concentration- and time-dependent manner, with IC_50_ values of 6.69 and 0.84 μM at 48 and 72 h, respectively (Figure 3A). Meanwhile, the long-term effects of **7** on K562 cells proliferation were determined with a colony formation assay in soft agar, an in vitro indicator of malignancy. As shown in Figure 3B,C, treatment of K562 cells with **7** (0–2 μM) for 12 days dose-dependently reduced the formation of colonies, indicating the antileukemia activity of **7**. Collectively, these results indicated that **7** inhibited the proliferation of K562 cells in vitro.

#### 2.2.3. Compound **7** Induced G0/G1 Phase Cell Cycle Arrest in K562 Cells

**Figure 4:**
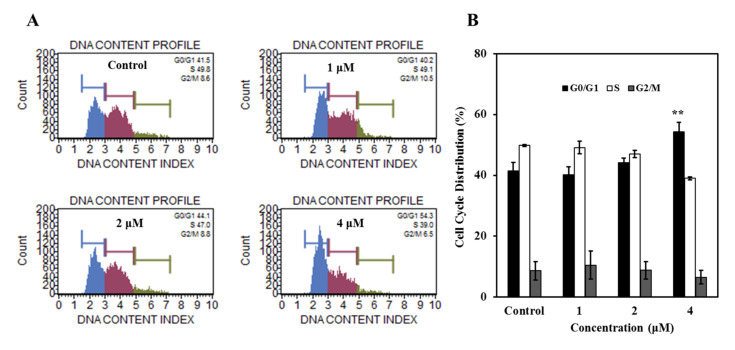
Compound **7** arrested cell cycle at G0/G1 phase. (**A**) Effect of compound **7** on cell cycle distribution. K562 cells after 36 h treatment with 0–4 μM of **7** were collected, washed, fixed, strained, and measured using Muse Cells Analyzer. (**B**) The histogram represented the cell cycle distributions in samples treated with or without **7**. Data are presented as mean ± SD of three independent experiments, ** *p* < 0.01, compared to the control.

To explore the underlying mechanisms leading to growth inhibition further, we analyzed the effect of **7** on the cell cycle distribution of K562 cells. The results showed that **7** induced moderate G0/G1 phase arrest in K562 cells at the concentration of 4 μM. In the control group, the cells in G0/G1 phase represented 41.5%, and it increased to 54.3% in the **7**-treated group. Correspondingly, the cells in the S phase were decreased after being treated by **7** (Figure 4A,B). It is worth mentioning that was totally different from BFA inducing G2/M phase cell cycle arrest in K562 cells [34]. Collectively, cell cycle arrest in G0/G1 phase may at least in part account for **7**-induced proliferation inhibition in K562 cells.

#### 2.2.4. Compound **7** Induced Caspase-Dependent Apoptosis in K562 Cells

**Figure 5:**
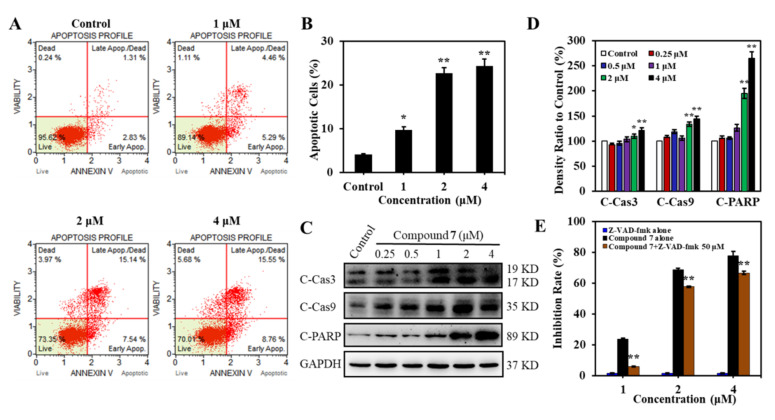
Compound **7** induced apoptosis in K562 cells. (**A**) Annexin V-PE/7-AAD staining in K562 cells incubated with 0–4 μM of **7** for 24 h was measured by Muse Cell Analyzer. (**B**) The bar graph depicted the percentage of apoptotic cells induced by **7**. Data are presented as mean ± SD for three independent experiments. * *p* < 0.05, ** *p* < 0.01, versus control. (**C**) Effect of **7** on apoptosis-related proteins. K562 cells were treated with vehicle or **7** at 0–4 μM for 36 h. Western blotting was performed to analyze the expression of C-Cas 3, C-Cas 9, and C-PARP. GAPDH was immunoblotted as a loading control. (**D**) Histograms show the relative abundance of the aimed bands to the control group. Data are presented as mean ± SD for three independent experiments. * *p* < 0.05, ** *p* < 0.01, versus control. (**E**) Pan caspase inhibitor Z-VAD-fmk significantly attenuated **7**-induced cell death. K562 cells were pretreated with or without Z-VAD-fmk for 1 h followed by incubation with various concentrations of **7** for 48 h, and then cells were subjected to MTT assay. Data are expressed as mean ± SD (*n* = 3). ** *p* < 0.01, versus **7** alone.

To determine whether the proliferative inhibition induced by **7** was attributed to apoptosis, K562 cells were treated with **7** at different concentrations for 24 h, followed by Annexin V-PE/7-AAD staining measured with flow cytometry. Compound **7** induced dose-dependent apoptosis with the apoptotic cells increased from 4.14% (in the control group) to 24.31% at the concentration of 4 μM (Figure 5A,B). The proapoptotic effect of **7** was also evidenced by the elevated apoptosis markers C-Cas 3, C-Cas 9, and C-PARP (Figure 5C,D). To further confirm whether caspases activation was involved in **7**-induced cell apoptosis, pan-caspase inhibitor Z-VAD-fmk was used to observe the attenuation of apoptotic cells. Consist with our hypothesis, **7**-stimulated apoptosis was attenuated significantly in the presence of Z-VAD-fmk (Figure 5E). These results demonstrated that compound **7** induced caspase-dependent apoptosis in K562 cells.

#### 2.2.5. Compound **7** Inactivated BCR-ABL and Affected Its Downstream Signaling Pathways in K562 Cells

**Figure 6:**
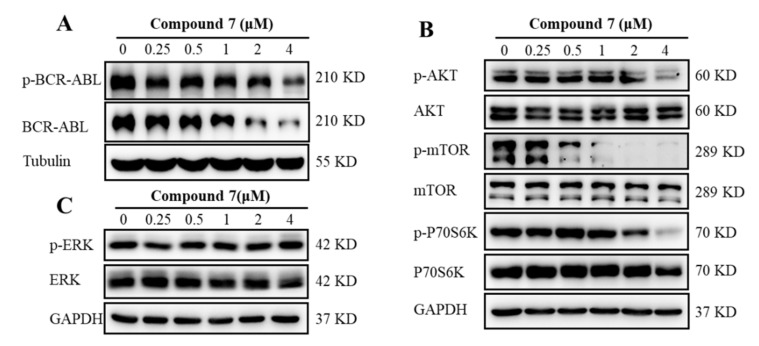
Compound **7** inhibited the activation of AKT/mTOR signaling pathway. (**A**) **7** induced the total and phosphorylated BCR-ABL inhibition. K562 cells were treated with indicated concentrations of **7** for 36 h, and the protein levels of BCR-ABL and p-BCR-ABL were subjected to Western blotting. (**B**) AKT/mTOR/S6K1 pathways were inactivated by **7**. Cells were treated as described before; then, the total and phosphorylated expression of AKT, mTOR, and p70S6K were evaluated by Western blotting. (**C**) Effect of **7** on ERK activation. K562 cells were treated as described above, and the protein levels of ERK and p-ERK were detected by Western blotting.

With the aim of obtaining insights into the potential mechanisms underlying **7**-induced inhibition of cell proliferation in K562 cells, the activation of BCR-ABL was first examined since CML cells were highly dependent on the presence of BCR-ABL [35]. The results suggested that **7** downregulated the total and phosphorylation levels of BCR-ABL (Figure 6A), which consisted of BFA reported as a functional inhibitor and degrader of BCR-ABL in our previous work [34]. The alternation of the key downstream signaling pathways of BCR-ABL was further explored, including AKT/mTOR/S6K1 and MAPK/ERK pathways, which were also important intracellular signaling pathways in tumorigenesis [36]. BFA (**1**) was also found to decrease the downstream signaling pathways of BCR-ABL, such as p-Akt and p-STAT5, while increased p-ERK in K562 cells [34]. AKT/mTOR/S6K1 pathway is a critical way for enhanced survival of BCR-ABL in leukemia cells, and many anticancer agents exert their anticancer effect by blocking this signal pathway and consequently enhancing apoptosis [37,38,39]. Therefore, the inactivation of key molecules in this pathway could be effective in CML treatment. For example, PI3K inhibitor LY294002 sensitized CML cells to nilotinib and increased apoptosis [40]. PI3K and mTOR inhibitor NVP-BEZ235 in combination with imatinib or nilotinib induced significant proliferation inhibition and apoptosis in BCR-ABL-positive cell lines [41]. The Western blot analysis indicated that **7** significantly inhibited the phosphorylation of AKT, thus leading to the downstream inhibition of the signaling molecules mTOR and p70S6K (Figure 6B). However, **7** showed no significant effect against the ERK1/2 signaling pathway (Figure 6C). Combined with the differences with **1** in cell cycle arrest and signal regulation, this implied that **7** was not a simple derivative of **1** and may have its own unique mode of action. These results suggested that **7** exhibited its antileukemia effect via at least partially inhibiting the activation of BCR-ABL and subsequently inactivating the downstream AKT/mTOR/S6K1 signaling pathway in K562 cells.

### 2.3. Molecular Modeling and Ligand Docking of Compound ***7*** into the Binding Site of ***1***

**Figure 7:**
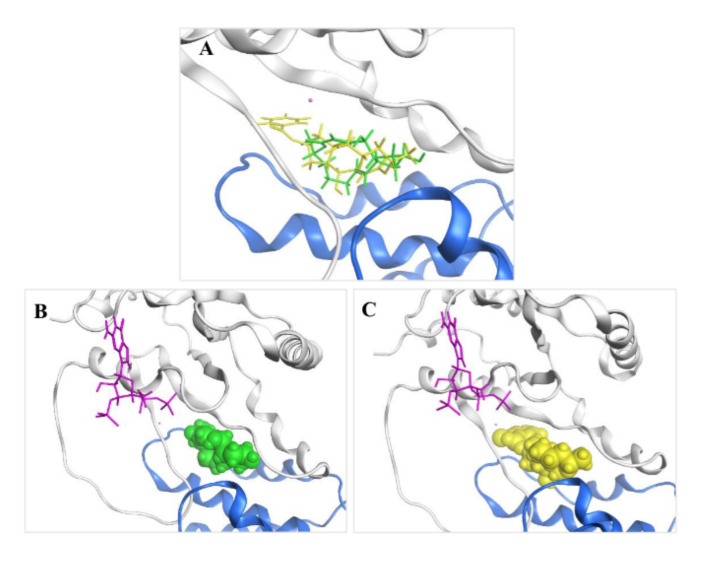
(**A**) Overlay of the docked pose of BFA (**1**, green) and BFA 7-*O*-2-chloro-4,5-difluorobenzoate (**7**, yellow) in the ARF1–GDP–GEF interface. (**B**) Crystal structure of **1** in the ARF1–GDP–GEF interface. (**C**) Model of **7** in the ARF1–GDP–GEF interface. Guanosine-3′-monophosphate-5′-diphosphate (GDP), purple; Mg^2+^ ion, pink; ARF1, gray; GEF, blue.

Given insights into the observation published by Renault et al. (PDB ID: 1R8Q) that the exquisite structural and chemical fit of **1** to the ARF–GDP-GEF interface accounted for its biological specificity [17], the structure of **7** was also investigated by molecular modeling to determine how well it would be tolerated in this pocket and whether it would fulfill the binding interactions necessary for inhibiting the GDP/GTP nucleotide exchange. Docking experiments were carried out with the X-ray crystal structure of the ARF1–GDP–Mg^2+^–BFA–ARNO complex [17] using the software Molecular Operating Environment (MOE). Thus, **7** was modeled into the binding site between ARF1 and ARNO, overlapping with the structure of **1**, which was then deleted (Figure 7A). The result showed that compound **7** was well tolerated in the active site of **1** (Figure 7B,C) and achieved a little higher docking score than **1**, indicating a good geometric fit in the binding pocket.

## 3. Materials and Methods

### 3.1. General Experimental Procedures

NMR spectra were recorded on a JEOL JEM-ECP NMR spectrometer. Chemical shifts *δ* are reported in ppm, using TMS as internal standard, and coupling constants (*J*) are in Hz. HRESIMS and ESIMS spectra were obtained from a Micromass Q-TOF spectrometer. Single-crystal data were measured on an Agilent Gemini Ultra diffractometer (Cu K*α* radiation). UPLC-MS was performed on Waters UPLC^®^ system (Waters Ltd., Milford, MA, USA) using a C18 column [(Waters Ltd.) ACQUITY UPLC^®^ BEH C18, 2.1 × 50 mm, 1.7 μm; 0.5 mL/min] and ACQUITY QDa ESIMS scan from 150 to 1000 Da. Silica gel (Qing Dao Hai Yang Chemical Group Co., Qingdao, China; 200–300 mesh) and Sephadex LH-20 (Amersham Biosciences, Amersham, UK) were used for column chromatography (CC). TLC silica gel plates (Yan Tai Zi Fu Chemical Group Co., Yantai, China; G60, F-254) were used for thin-layer chromatography. Semi-preparative HPLC was operated on a Waters 1525 system using a semi-preparative C18 (Kromasil, 5 μm, 10 × 250 mm) column equipped with a Waters 2996 photodiode array detector, at a flow rate of 2.0 mL/min.

### 3.2. Fungal Material

The fungal strain *Penicillium* sp. (HS-N-29) was isolated from a piece of fresh tissue from the inner part of the medicinal mangrove *Acanthus ilicifolius* collected from the South China Sea. The strain was identified according to a molecular biological protocol by DNA amplification and sequencing of the ITS region as described in the literature [42]. The fungal strain was identified as *Penicillium* sp. with the accession number MW178203.

### 3.3. Fermentation, Extraction, and Isolation

The fungal strain was cultivated in 50 L of PDB medium (200.0 g of potato, 20.0 g of glucose in 1 L of seawater, in 500 mL Erlenmeyer flasks, each containing 250 mL of culture broth) at 28 °C with shaking for 2 weeks. The fungal culture was filtered and extracted three times with EtOAc. The EtOAc extract was combined and concentrated under vacuum to afford a dry crude extract (55.0 g). This extract was fractionated into five fractions (Fr.1–Fr.5) by silica gel VLC with a stepwise gradient of petroleum ether-EtOAc. Subsequently, Fr.3 (25.0 g) was separated into four subfractions (Fr.3-1–Fr.3-4) by silica gel CC (200–300 mesh) with a step gradient of petroleum ether-EtOAc from 3:1 to 0:1 (*v*/*v*). Fr.3-2 was further purified by semipreparative HPLC (70% MeCN–H_2_O) and then recrystallized to produce **1** (2.3 g, Figures S1–S3).

### 3.4. General Synthetic Methods for Compounds ***2**–**16***

Benzoic acid-derived reagent (1–2 equivalent) was added to a solution of **1** (50.0 mg, 0.18 mmol), DMAP (21.8 mg, 0.18 mmol), and EDCl (110.7 mg, 0.71 mmol) in 15 mL dry dichloromethane (DCM). The reaction mixture was stirred at 45 °C, and the progress of the reaction was monitored by silica gel TLC and UPLC-MS. After 1–3 h, the reaction mixture was quenched with water and diluted with DCM. The organic layer was separated, and the solvent was removed under reduced pressure. The residue was purified by silica gel CC followed by semi-preparative HPLC to yield unreacted **1** and derivatives **2**–**16** (Figures S4–S48).

**Brefeldin A 7-*O*-benzoate (2)**: Known compound. Colorless oil; yield 11%; ^1^H NMR (400 MHz, CDCl_3_) *δ* 8.03–7.98 (2H, overlapped), 7.56 (1H, m), 7.47–7.43 (2H, overlapped), 7.37 (1H, dd, *J* = 15.7, 3.1 Hz), 5.94 (1H, dd, *J* = 15.7, 1.9 Hz), 5.73 (1H, m), 5.40 (1H, m), 5.26 (1H, dd, *J* = 15.2, 9.0 Hz), 4.86 (1H, m), 4.17 (1H, m), 2.53–2.33 (3H, overlapped), 2.06–1.96 (2H, overlapped), 1.92 (1H, m), 1.88–1.80 (2H, overlapped), 1.77–1.70 (2H, overlapped), 1.53 (1H, m), 1.26 (3H, d, *J* = 6.2 Hz), 0.96 (1H, m); ^13^C NMR (100 MHz, CDCl_3_) *δ* 166.3 (C = O), 166.3 (C = O), 151.6 (CH), 136.1 (CH), 133.1 (CH), 131.1 (CH), 130.6 (C), 129.7 (CH × 2), 128.5 (CH × 2), 117.9 (CH), 76.1 (CH), 76.0 (CH), 71.9 (CH), 52.6 (CH), 44.1 (CH), 40.3 (CH_2_), 38.9 (CH_2_), 34.2 (CH_2_), 32.0 (CH_2_), 26.8 (CH_2_), 21.0 (CH_3_). ESIMS *m*/*z* 407.4 [M + Na]^+^.

**Brefeldin A 4,7-*O*-dibenzoate (3)**: Known compound. Colorless oil; yield 43%; ^1^H NMR (500 MHz, CDCl_3_) *δ* 8.09–8.00 (4H, overlapped), 7.61–7.54 (2H, overlapped), 7.48–7.42 (4H, overlapped), 7.38 (1H, dd, *J* = 15.6, 3.4 Hz), 5.85–5.75 (2H, overlapped), 5.58 (1H, ddd, *J* = 10.4, 3.4, 1.9 Hz), 5.42 (1H, m), 5.34 (1H, dd, *J* = 15.2, 9.5 Hz), 4.87 (1H, m), 2.64 (1H, m), 2.48 (1H, m), 2.38 (1H, m), 2.28 (1H, m), 2.03 (1H, m), 1.92–1.84 (3H, overlapped), 1.81 (1H, m), 1.75 (1H, m), 1.55 (1H, m), 1.25 (3H, d, *J* = 6.1 Hz), 0.97 (1H, m); ^13^C NMR (100 MHz, CDCl_3_) *δ* 166.1 (C = O), 165.8 (C = O), 165.5 (C = O), 147.3 (CH), 135.9 (CH), 133.6 (CH), 133.1 (CH), 131.4 (CH), 130.6 (C), 129.9 (CH × 2), 129.7 (CH × 2), 129.6 (C), 128.7 (CH × 2), 128.6 (CH × 2), 118.7 (CH), 77.0 (CH), 76.2 (CH), 72.0 (CH), 50.5 (CH), 44.4 (CH), 40.3 (CH_2_), 38.8 (CH_2_), 34.2 (CH_2_), 32.0 (CH_2_), 26.7 (CH_2_), 20.9 (CH_3_). HRESIMS *m*/*z* 489.2263 [M + H]^+^ (calcd. for C_30_H_33_O_6_^+^, 489.2272).

**Brefeldin A 4-*O*-benzoate (4)**: Colorless oil; yield 33%; ^1^H NMR (400 MHz, CDCl_3_) *δ* 8.09–8.04 (2H, overlapped), 7.59 (1H, m), 7.49–7.42 (2H, overlapped), 7.35 (1H, dd, *J* = 15.7, 3.2 Hz), 5.82–5.68 (2H, overlapped), 5.50 (1H, ddd, *J* = 10.5, 3.3, 1.8 Hz), 5.34 (1H, dd, *J* = 15.2, 9.5 Hz), 4.84 (1H, m), 4.33 (1H, m), 2.50 (1H, m), 2.30 (1H, m), 2.24 (1H, m), 2.09–1.94 (2H, overlapped), 1.88–1.79 (2H, overlapped), 1.76–1.65 (2H, overlapped), 1.61–1.47 (2H, overlapped), 1.23 (3H, d, *J* = 6.2 Hz), 0.94 (1H, m); ^13^C NMR (100 MHz, CDCl_3_) *δ* 165.8 (C = O), 165.6 (C = O), 147.4 (CH), 136.6 (CH), 133.5 (CH), 130.8 (CH), 129.8 (CH × 2), 129.7(C), 128.7 (CH × 2), 118.4 (CH), 77.0 (CH), 72.6 (CH), 72.0 (CH), 49.9 (CH), 44.5 (CH), 43.3 (CH_2_), 41.2 (CH_2_), 34.2 (CH_2_), 31.9 (CH_2_), 26.8 (CH_2_), 20.9 (CH_3_). HRESIMS *m*/*z* 385.2004 [M + H]^+^ (calcd. for C_23_H_29_O_5_^+^, 385.2010).

**Brefeldin A 7-*O*-(2,3,4)-trifluorobenzoate (5)**: Colorless oil; yield 10%; ^1^H NMR (500 MHz, CDCl_3_) *δ* 7.70 (1H, m), 7.36 (1H, dd, *J* = 15.7, 3.2 Hz), 7.04 (1H, m), 5.92 (1H, dd, *J* = 15.8, 2.0 Hz), 5.73 (1H, m), 5.43 (1H, m), 5.27 (1H, dd, *J* = 15.2, 9.2 Hz), 4.87 (1H, m), 4.17 (1H, m), 2.51–2.35 (3H, overlapped), 2.06–1.95 (2H, overlapped), 1.94–1.80 (3H, overlapped), 1.79–1.72 (2H, overlapped), 1.53 (1H, m), 1.26 (3H, d, *J* = 6.3 Hz), 0.95 (1H, m); ^13^C NMR (125 MHz, CDCl_3_) *δ* 166.2 (C = O), 162.6 (C = O), 151.4 (CH), 135.9 (CH), 131.3 (CH), 126.4 (C), 126.4 (C), 126.3 (C), 126.3 (C), 118.0 (CH), 112.3 (CH), 112.2 (CH), 77.2 (CH), 76.0 (CH), 71.9 (CH), 52.5 (CH), 44.1 (CH), 40.2 (CH), 39.0 (CH_2_), 34.3 (CH_2_), 32.0 (CH_2_), 26.8 (CH_2_), 21.0 (CH_3_). HRESIMS *m*/*z* 439.1726 [M + H]^+^ (calcd. for C_23_H_26_F_3_O_5_^+^, 439.1727).

**Brefeldin A 4-*O*-(2,3,4)-trifluorobenzoate (6)**: Colorless oil; yield 28%; ^1^H NMR (500 MHz, CDCl_3_) *δ* 7.75 (1H, m), 7.31 (1H, dd, *J* = 15.7, 3.4 Hz), 7.07 (1H, m), 5.80–5.69 (2H, overlapped), 5.51 (1H, ddd, *J* = 10.6, 3.5, 1.9 Hz), 5.34 (1H, dd, *J* = 15.2, 9.6 Hz), 4.86 (1H, m), 4.35 (1H, m), 2.49 (1H, m), 2.38–2.22 (2H, overlapped), 2.08–1.96 (2H, overlapped), 1.89–1.82 (2H, overlapped), 1.77–1.67 (2H, overlapped), 1.58–1.50 (2H, overlapped), 1.25 (3H, d, *J* = 6.2 Hz), 0.95 (1H, m); ^13^C NMR (125 MHz, CDCl_3_) *δ* 165.7 (C = O), 162.0 (C = O), 146.6 (CH), 136.4 (CH), 130.9 (CH), 126.5 (C), 126.5 (C), 126.5 (C), 126.4 (C), 118.9 (CH), 112.5 (CH), 112.4 (CH), 78.0 (CH), 72.6 (CH), 72.1 (CH), 49.8 (CH), 44.5 (CH), 43.3 (CH_2_), 41.3 (CH_2_), 34.2 (CH_2_), 32.0 (CH_2_), 26.7 (CH_2_), 20.9 (CH_3_). HRESIMS *m*/*z* 439.1723 [M + H]^+^ (calcd. for C_23_H_26_F_3_O_5_^+^, 439.1727).

**Brefeldin A 7-*O*-2-chloro-4,5-difluorobenzoate (7)**: Colorless oil; yield 15%; ^1^H NMR (400 MHz, CDCl_3_) *δ* 7.69 (1H, dd, *J* = 10.4, 8.4 Hz), 7.34 (1H, dd, *J* = 15.7, 3.2 Hz), 7.29 (1H, dd, *J* = 9.8, 6.9 Hz), 5.92 (1H, dd, *J* = 15.7, 1.9 Hz), 5.73 (1H, m), 5.38 (1H, m), 5.24 (1H, dd, *J* = 15.2, 8.8 Hz), 4.85 (1H, m), 4.15 (1H, ddd, *J* = 9.2, 3.1, 1.9 Hz), 2.53–2.31 (3H, overlapped), 2.03–1.91 (3H, overlapped), 1.90–1.80 (2H, overlapped), 1.78–1.67 (2H, overlapped), 1.53 (1H, m), 1.25 (3H, d, *J* = 6.3 Hz), 0.86 (1H, m); ^13^C NMR (100 MHz, CDCl_3_) *δ* 166.3 (C = O), 163.5 (C = O), 151.5 (CH), 135.8 (CH), 131.4 (CH), 120.7 (C), 120.7 (C), 120.6 (CH), 120.5 (C), 120.5 (C), 120.4 (CH), 118.0 (CH), 77.4 (CH), 75.9 (CH), 71.9 (CH), 52.3 (CH), 44.1 (CH), 40.2 (CH_2_), 38.9 (CH_2_), 34.2 (CH_2_), 31.9 (CH_2_), 26.7 (CH_2_), 20.9 (CH_3_). HRESIMS *m*/*z* 455.1429 [M + H]^+^ (calcd. for C_23_H_26_ClF_2_O_5_^+^, 455.1431).

**Brefeldin A 4-*O*-2-chloro-4,5-difluorobenzoate (8)**: Colorless oil; yield 30%; ^1^H NMR (400 MHz, CDCl_3_) *δ* 7.77 (1H, dd, *J* = 10.3, 8.3 Hz), 7.33 (1H, m), 7.28 (1H, d, *J* = 12.4 Hz), 5.82–5.63 (2H, overlapped), 5.48 (1H, ddd, *J* = 10.5, 3.5, 1.8 Hz), 5.31 (1H, dd, *J* = 15.2, 9.5 Hz), 4.85 (1H, m), 4.32 (1H, m), 2.47 (1H, m), 2.33 (1H, m), 2.22 (1H, m), 2.06–1.94 (2H, overlapped), 1.91–1.80 (2H, overlapped), 1.75–1.65 (2H, overlapped), 1.57–1.47 (2H, overlapped), 1.23 (3H, d, *J* = 6.3 Hz), 0.93 (1H, m); ^13^C NMR (100 MHz, CDCl_3_) *δ* 165.6 (C = O), 162.6 (C = O), 146.5 (CH), 136.3 (CH), 130.9 (CH), 120.9 (C), 120.8 (C), 120.8 (CH), 120.7 (C), 120.6 (C), 120.6 (CH), 118.8 (CH), 78.3 (CH), 72.4 (CH), 72.1 (CH), 49.6 (CH), 44.5 (CH), 43.3 (CH_2_), 41.2 (CH_2_), 34.1 (CH_2_), 31.9 (CH_2_), 26.7 (CH_2_), 20.9 (CH_3_). HRESIMS *m*/*z* 455.1426 [M + H]^+^ (calcd. for C_23_H_26_ClF_2_O_5_^+^, 455.1431).

**Brefeldin A 4,7-*O*-di-2-chloro-4,5-difluorobenzoate (9)**: Colorless oil; yield 35%; ^1^H NMR (400 MHz, CDCl_3_) *δ* 7.78 (1H, dd, *J* = 10.3, 8.3 Hz), 7.69 (1H, dd, *J* = 10.3, 8.3 Hz), 7.37–7.26 (3H, overlapped), 5.86–5.70 (2H, overlapped), 5.55 (1H, ddd, *J* = 10.1, 3.6, 1.8 Hz), 5.40 (1H, m), 5.29 (1H, dd, *J* = 15.4, 9.2 Hz), 4.88 (1H, m), 2.60 (1H, m), 2.50 (1H, m), 2.41–2.24 (2H, overlapped), 2.03 (1H, m), 1.96–1.70 (5H, overlapped), 1.54 (1H, m), 1.25 (3H, d, *J* = 6.2 Hz), 0.96 (1H, m); ^13^C NMR (100 MHz, CDCl_3_) *δ* 165.5 (C = O), 163.4 (C = O), 162.6 (C = O), 146.0 (CH), 135.3 (CH), 131.9 (CH), 121.0 (C), 121.0 (C), 120.9 (CH), 120.8 (C), 120.8 (C), 120.7 (C), 120.7 (C), 120.7 (CH), 120.6 (CH), 120.5 (C), 120.5 (C), 120.4 (CH), 119.3 (CH), 78.1 (CH), 77.3 (CH), 72.1 (CH), 50.0 (CH), 44.3 (CH), 40.2 (CH_2_), 38.8 (CH_2_), 34.2 (CH_2_), 31.9 (CH_2_), 26.6 (CH_2_), 20.9 (CH_3_). HRESIMS *m*/*z* 629.1117 [M + H]^+^ (calcd. for C_30_H_27_Cl_2_F_4_O_6_^+^, 629.1115).

**Brefeldin A 7-*O*-(4)-chlorobenzoate (10)**: Colorless oil; yield 10%; ^1^H NMR (600 MHz, CDCl_3_) *δ* 7.96–7.92 (2H, overlapped), 7.43–7.40 (2H, overlapped), 7.37 (1H, dd, *J* = 15.6, 3.0 Hz), 5.93 (1H, d, *J* = 15.5 Hz), 5.74 (1H, m), 5.39 (1H, m), 5.24 (1H, dd, *J* = 15.2, 9.2 Hz), 4.87 (1H, m), 4.18 (1H, d, *J* = 9.6 Hz), 2.51–2.33 (3H, overlapped), 2.05–1.96 (2H, overlapped), 1.91 (1H, m), 1.88–1.81 (2H, overlapped), 1.78–1.70 (2H, overlapped), 1.53 (1H, m), 1.27 (3H, d, *J* = 6.4 Hz), 0.96 (1H, m); ^13^C NMR (150 MHz, CDCl_3_) *δ* 166.2 (C = O), 165.4 (C = O), 151.4 (CH), 139.6 (C), 136.0 (CH), 131.3 (CH), 131.1 (CH × 2), 129.0 (C), 128.9 (CH × 2), 118.0 (CH), 76.4 (CH), 76.0 (CH), 71.9 (CH), 52.5 (CH), 44.1 (CH), 40.3 (CH_2_), 38.9 (CH_2_), 34.3 (CH_2_), 32.0 (CH_2_), 26.8 (CH_2_), 21.0 (CH_3_). HRESIMS *m*/*z* 419.1620 [M + H]^+^ (calcd. for C_23_H_28_ClO_5_^+^, 419.1620).

**Brefeldin A 4-*O*-(4)-chlorobenzoate (11)**: Colorless oil; yield 30%; ^1^H NMR (600 MHz, CDCl_3_) *δ* 8.02–7.95 (2H, overlapped), 7.45–7.40 (2H, overlapped), 7.32 (1H, dd, *J* = 15.7, 3.3 Hz), 5.77–5.67 (2H, overlapped), 5.48 (1H, ddd, *J* = 10.5, 3.3, 1.8 Hz), 5.32 (1H, dd, *J* = 15.2, 9.6 Hz), 4.84 (1H, m), 4.30 (1H, m), 2.47 (1H, m), 2.31 (1H, m), 2.23 (1H, m), 2.05–1.92 (2H, overlapped), 1.88–1.81 (2H, overlapped), 1.71 (1H, m), 1.66 (1H, m), 1.56–1.47 (2H, overlapped), 1.22 (3H, d, *J* = 6.2 Hz), 0.92 (1H, m); ^13^C NMR (150 MHz, CDCl_3_) *δ* 165.8 (C = O), 164.7 (C = O), 147.2 (CH), 140.0 (C), 136.5 (CH), 131.2 (CH × 2), 130.8 (CH), 129.0 (CH × 2), 128.1 (C), 118.4 (CH), 77.3 (CH), 72.4 (CH), 72.1 (CH), 49.8 (CH), 44.4 (CH), 43.3 (CH_2_), 41.1 (CH_2_), 34.1 (CH_2_), 31.9 (CH_2_), 26.7 (CH_2_), 20.9 (CH_3_). HRESIMS *m*/*z* 419.1626 [M + H]^+^ (calcd. for C_23_H_28_ClO_5_^+^, 419.1620).

**Brefeldin A 4,7-*O*-di-(4)-chlorobenzoate (12)**: Colorless oil; yield 36%; ^1^H NMR (600 MHz, CDCl_3_) *δ* 8.03–7.88 (4H, overlapped), 7.46–7.36 (4H, overlapped), 7.34 (1H, dd, *J* = 15.7, 3.3 Hz), 5.84–5.72 (2H, overlapped), 5.55 (1H, ddd, *J* = 10.4, 3.4, 1.8 Hz), 5.37 (1H, m), 5.28 (1H, dd, *J* = 15.2, 9.6 Hz), 4.84 (1H, m), 2.60 (1H, m), 2.44 (1H, m), 2.32 (1H, m), 2.22 (1H, m), 2.01 (1H, m), 1.92–1.80 (3H, overlapped), 1.81–1.68 (2H, overlapped), 1.52 (1H, m), 1.22 (3H, d, *J* = 6.1 Hz), 0.88 (1H, m); ^13^C NMR (125 MHz, CDCl_3_) *δ* 165.5 (C = O), 165.1 (C = O), 164.5 (C = O), 146.8 (CH), 139.9 (C), 139.4 (C), 135.5 (CH), 131.5 (CH), 131.1 (CH × 2), 130.9 (CH × 2), 128.9 (CH × 2), 128.8 (C), 128.7 (CH × 2), 127.9 (C), 118.6 (CH), 77.0 (CH), 76.3 (CH), 72.0 (CH), 50.1 (CH), 44.1 (CH), 40.1 (CH_2_), 38.5 (CH_2_), 34.1 (CH_2_), 31.8 (CH_2_), 26.5 (CH_2_), 20.8 (CH_3_). HRESIMS *m*/*z* 557.1486 [M + H]^+^ (calcd. for C_30_H_31_Cl_2_O_6_^+^, 557.1492).

**Brefeldin A 7-*O*-2-chloro-4-fluorobenzoate (13)**: Colorless oil; yield 12%; ^1^H NMR (400 MHz, CDCl_3_) *δ* 7.85 (1H, dd, *J* = 8.8, 6.1Hz), 7.35 (1H, dd, *J* = 15.7, 3.2 Hz), 7.19 (1H, dd, *J* = 8.5, 2.5 Hz), 7.03 (1H, ddd, *J* = 8.8, 7.6, 2.5 Hz), 5.92 (1H, dd, *J* = 15.7, 2.0 Hz), 5.73 (1H, m), 5.41 (1H, m), 5.26 (1H, dd, *J* = 15.2, 8.9 Hz), 4.87 (1H, m), 4.17 (1H, ddd, *J* = 9.4, 3.2, 2.0 Hz), 2.52–2.33 (3H, overlapped), 2.04–1.80 (5H, overlapped), 1.78–1.71 (2H, overlapped), 1.52 (1H, m), 1.28–1.24 (3H, d, *J* = 6.3 Hz), 0.95 (1H, m); ^13^C NMR (100 MHz, CDCl_3_) *δ* 166.3 (C = O), 164.5 (C = O), 151.4 (CH), 135.9 (CH), 133.7 (C), 133.6 (C), 131.3 (CH), 118.9 (C), 118.6 (CH), 118.0 (CH), 114.4 (CH), 114.2 (CH), 76.9 (CH), 76.0 (CH), 71.9 (CH), 52.5 (CH), 44.2 (CH), 40.2 (CH_2_), 38.9 (CH_2_), 34.3 (CH_2_), 32.0 (CH_2_), 26.8 (CH_2_), 21.0 (CH_3_); HRESIMS *m*/*z* 437.1522 [M + H]^+^ (calcd. for C_23_H_27_ClFO_5_^+^, 437.1526).

**Brefeldin A 4-*O*-2-chloro-4-fluorobenzoate (14)**: Colorless oil; yield 30%; ^1^H NMR (600 MHz, CDCl_3_) *δ* 7.95 (1H, dd, *J* = 8.8, 6.1 Hz), 7.31 (1H, dd, *J* = 15.7, 3.4 Hz), 7.22 (1H, dd, *J* = 8.5, 2.5 Hz), 7.07 (1H, m), 5.81–5.70 (2H, overlapped), 5.51 (1H, ddd, *J* = 10.5, 3.4, 1.8 Hz), 5.32 (1H, dd, *J* = 15.2, 9.6 Hz), 4.87 (1H, m), 4.35 (1H, m), 2.49 (1H, m), 2.32 (1H, m), 2.24 (1H, m), 2.08–1.96 (2H, overlapped), 1.89–1.82 (2H, overlapped), 1.80–1.68 (2H, overlapped), 1.57–1.52 (2H, overlapped), 1.25 (3H, d, *J* = 6.3 Hz), 0.94 (1H, m); ^13^C NMR (150 MHz, CDCl_3_) *δ* 165.8 (C = O), 163.7 (C = O), 146.9 (CH), 136.4 (CH), 133.9 (C), 133.8 (C), 130.9 (CH), 119.1 (CH), 118.9 (C), 118.8 (CH), 114.5 (CH), 114.3 (CH), 77.8 (CH), 72.6 (CH), 72.1 (CH), 49.8 (CH), 44.5 (CH), 43.4 (CH_2_), 41.3 (CH_2_), 34.2 (CH_2_), 31.9 (CH_2_), 26.7 (CH_2_), 20.9 (CH_3_). HRESIMS *m*/*z* 437.1530 [M + H]^+^ (calcd. for C_23_H_27_ClFO_5_^+^, 437.1526).

**Brefeldin A 4,7-*O*-di-2-chloro-4-fluorobenzoate (15)**: Colorless oil; yield 36%; ^1^H NMR (600 MHz, CDCl_3_) *δ* 7.94 (1H, dd, *J* = 8.8, 6.1 Hz), 7.85 (1H, dd, *J* = 8.8, 6.1 Hz), 7.32 (1H, dd, *J* = 15.7, 3.5 Hz), 7.26–7.17 (2H, overlapped), 7.08–7.01 (2H, overlapped), 5.84–5.73 (2H, overlapped), 5.56 (1H, ddd, *J* = 10.1, 3.5, 1.8 Hz), 5.42 (1H, m), 5.30 (1H, m), 4.88 (1H, m), 2.60 (1H, m), 2.49 (1H, m), 2.40–2.28 (2H, overlapped), 2.02 (1H, m), 1.95–1.83 (3H, overlapped), 1.80 (1H, m), 1.74 (1H, m), 1.55 (1H, m), 1.25 (3H, d, *J* = 6.2 Hz), 0.97 (1H, m); ^13^C NMR (150 MHz, CDCl_3_) *δ* 165.6 (C = O), 164.5 (C = O), 163.7 (C = O), 146.5 (CH), 135.6 (CH), 133.9 (C), 133.9 (C), 133.7 (C), 133.6 (C), 131.7 (CH), 119.1 (CH × 2), 118.9 (CH), 118.9 (C), 118.7 (C), 114.5 (CH), 114.4 (CH), 114.4 (CH), 114.2 (CH), 77.7 (CH), 76.9 (CH), 72.1 (CH), 50.2 (CH), 44.4 (CH), 40.2 (CH_2_), 38.9 (CH_2_), 34.2 (CH_2_), 31.9 (CH_2_), 26.6 (CH_2_), 20.9 (CH_3_). HRESIMS *m*/*z* 593.1309 [M + H]^+^ (calcd. for C_30_H_29_Cl_2_F_2_O_6_^+^, 593.1304).

**Brefeldin A 7-*O*-(2,4,6)-trichlorobenzoate (16)**: Colorless oil; yield 15%; ^1^H NMR (500 MHz, CDCl_3_) *δ* 7.37–7.29 (3H, overlapped), 5.90 (1H, dd, *J* = 15.7, 1.9 Hz), 5.71 (1H, m), 5.46 (1H, m), 5.23 (1H, dd, *J* = 15.3, 8.5Hz), 4.84 (1H, m), 4.14 (1H, m), 2.46–2.37 (3H, overlapped), 2.05 (1H, d, *J* = 5.0 Hz), 2.01–1.90 (3H, overlapped), 1.87–1.78 (3H, overlapped), 1.73 (1H, m), 1.52 (1H, m), 1.25 (3H, d, *J* = 6.3 Hz), 0.92 (1H, m); ^13^C NMR (100 MHz, CDCl_3_) *δ* 166.3 (C = O), 163.8 (C = O), 151.4 (CH), 136.2 (C), 135.9 (CH), 132.6 (CH), 132.3 (C), 131.2 (CH), 128.2 (CH), 128.2 (C × 2),117.9 (CH), 78.0 (CH), 75.9 (CH), 71.9 (CH), 52.3 (CH), 44.1 (CH), 40.0 (CH_2_), 38.6 (CH_2_), 34.2 (CH_2_), 31.9 (CH_2_), 26.8 (CH_2_), 21.0 (CH_3_). HRESIMS *m*/*z* 487.0837 [M + H]^+^ (calcd. for C_23_H_26_Cl_3_O_5_^+^, 487.0840).

### 3.5. Biological Assays

#### 3.5.1. Reagents

Antibodies against AKT, phosphorylated AKT at S473, ERK1/2, phosphorylated ERK1/2 (T202/Y204), mTOR, phosphorylated mTOR (S2481), p70S6K, phosphorylated p70S6K (T421/S424), cleaved caspase-3 (C-Cas3), cleaved caspase-9 (C-Cas9), and cleaved PARP (C-PARP) were purchased from Cell Signaling Technology (Boston, MA, USA). Antibodies against GAPDH and Tubulin were obtained from Huaan Biotechnology Co., Ltd. (Hangzhou, China). Z-VAD-fmk was obtained from Selleck Chemicals (Houston, TX, USA). Muse™ Cell Cycle Kit and Muse^®^ Annexin V & Dead Cell Kit were purchased from Millipore (Billerica, MA, USA). Other reagents were purchased from Beyotime Biotechnology, Shanghai, China.

#### 3.5.2. Cell Lines and Cell Culture

Human chronic myelogenous leukemia cell line K562 was purchased from Shanghai Cell Bank, Chinese Academy of Sciences, and cells were maintained in Iscove’s Modified Dulbecco’s Medium (GIBCO, Grand Island, NY, USA) with 10% fetal bovine serum (FBS) and 1% penicillin/streptomycin in a humidified incubator at 37 °C under 5% CO_2_.

#### 3.5.3. Cell Proliferation Assay

The 3-(4,5-dimethylthiazol-2-yl)-2,5-diphenyltetrazolium bromide (MTT) assay was performed to determine cell viability. Briefly, K562 cells were seeded in 96-well plates and treated for the indicated time with various concentrations of compound 7. Then, 20 μL of MTT reagent was added to each well and further incubated for 4 h. Next, the formazan product was dissolved with acidic isopropanol (100 μL) incubated overnight. Finally, the absorbance was measured at 570 nm using a SpectraMax^®^ i3x multi-mode microplate reader (Molecular Devices, San Jose, CA, USA). Then, cells viability was calculated.

#### 3.5.4. Soft Agar Colony Formation Assay

Soft agar colony formation assay was performed as described previously [43]. In brief, 6-well plate was first coated with 1.2% agarose with an equal volume of 2× Dulbecco’s Modified Eagle’s Medium containing 20% fetal bovine serum, then K562 cells (2000/well) were mixed with the culture medium containing 0.4% agarose and indicated concentration of compound **7**, and then the mixture immediately overlaid on the pre-coated plates. The culture medium was changed every 3 days. After incubation for 12 d at 37 °C, cells were stained with 0.005% crystal violet, and colonies consisting of 50 or more cells were counted.

#### 3.5.5. Cell Cycle Analysis

K562 cells were seeded in 6-well plates (3 × 10^5^ cells/well) and treated with different concentrations of compound **7** for 36 h; the control group was treated with the same diluted DMSO. Then, cells were harvested, washed with ice-cold phosphate-buffered saline (PBS), and fixed in 70% cold ethanol overnight at −20 °C. Fixed cells were then collected, washed, and stained with 200 μL of Muse™ cell cycle reagent for 30 min in the dark at room temperature. The cell cycle distribution was immediately analyzed by Muse Cell Analyzer (Millipore, Billerica, MA, USA).

#### 3.5.6. Cell Apoptosis Analysis

Apoptosis was determined by Annexin V-PE/7-AAD staining [44]. After treatment with compound **7** for 36 h, cells were collected, centrifuged, and washed with ice-cold PBS. Then cells were resuspended in 100 μL Iscove’s Modified Dulbecco’s Medium with 1% FBS and stained with Muse^®^ Annexin V & Dead Cell Kit (Millipore, Billerica, MA, USA) for 20 min in the dark at room temperature and finally analyzed by Muse Cell Analyzer (Millipore, Billerica, MA, USA).

#### 3.5.7. Western Blotting Assay

Cells were seeded in 6-well plates (3 × 10^5^ cells/well) and incubated with compound **7** for the indicated time. Then, cells were collected, washed, suspended, and lysed in loading buffer (0.125 M Tris-HCl, 5% 2-mMercaptoethanol, 30 mg/mL sodium dodecyl sulfate (SDS), 10% glycerol, 0.5 mg/mL bromophenol blue) for 40 min at 4 °C. Proteins were separated by electrophoresis on 6–12% SDS-polyacrylamide gels and transferred to NC membranes (Millipore, Billerica, MA, USA). The membrane was blocked in 5% skim milk for 1 h and incubated with corresponding primary antibodies overnight at 4 °C, and subsequently, the membranes were incubated with HRP-secondary antibody at room temperature for 1 h and finally detected by Tanon 5200 (Tanon, Beijing, China).

#### 3.5.8. Statistical Analysis

The results shown in this study are represented as the mean values ± SD. Comparisons between the groups were assessed by Student’s *t*-test, and *p* < 0.05 was defined as statistically significant.

### 3.6. Solubility Measurement

Ten milligrams of **1** and **7** was added into the 15 mL centrifuge tubes, then 10 mL of water was added, and the tubes were shaken on a vortexes (Shanghai Qite Analytical Instrument Co., Shanghai, China; QT-2) at 1000 rpm for 30 min at room temperature. Subsequently, the tubes were ultrasonic dissolved for a further 3 h in an ultrasonic cleaner (Kunshan Ultrasonic Instrument Co., Kunshan, China; KQ-300DE). After completion of dissolution, the suspension was centrifuged at 15 000 rpm (Thermo Fisher, Waltham, MA, USA, Sorvall Legend Micro 17) for 10 min to precipitate undissolved particles. Then, the solid and liquid phases were separated, and the undissolved solid was dried and weighed.

### 3.7. Molecular Modeling and Protein–Ligand Docking

The crystal structure of **1** in the interface between ARF1-GDP and its GEF protein ARNO was downloaded from the Protein Data Bank (PDB ID: 1R8Q) [13]. The structure of **7** was selected for docking, followed by structure optimization with the software MOE (Chemical Computing Group ULC, Montreal, QC, Canada). The target proteins were performed for protonation with the Protonate 3D function in MOE and expulsion of water. The 3D structure of **7** was constructed with drawing software ChemDraw & Chem3D, and MM2 minimized in Chem3D prior to molecular docking. Then, **7** was docked in the binding pocket overlapped with **1**. Molecular docking with the Rigid Receptor parameter was conducted within MOE.

## 4. Conclusions

In this study, a series of derivatives of **1** were designed and synthesized, and their inhibitory effects against K562 cells were evaluated. The SARs studies revealed that the monoester derivatives exhibited stronger cytotoxic activity than those of diester derivatives. Moreover, the introduction of halogen atoms on the benzene rings showed a certain improvement in potency. The results exhibited that compound **7** showed great potential cytotoxic activity on K562 cells compared to other tested compounds, while the effect was slightly weaker than **1**. Further investigations indicated that **7** inhibited the growth of K562 cells, arrested cell cycle, and induced apoptosis. Additionally, the mechanism underlying **7**-induced cell proliferation inhibition was partially related to the inactivation of the BCR-ABL signaling pathway. The molecular models indicated that **7** was well tolerated in the interface between the ARF and ARNO proteins. In summary, the current results demonstrated that **7** has great promise against BCR-ABL positive cells and may serve as an efficacious antileukemia agent.

## Figures and Tables

**Table 1:** Cytotoxic activity of **1** and its derivatives **2**–**16**.

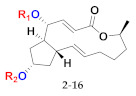					
Compounds		IC_50_ (μM) ^a^	Compounds		IC_50_ (μM) ^a^
Structure	Substituents	K562	Structure	Substituents	K562
brefeldin A	**1** R_1_ = R_2_ = H	0.24	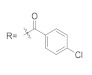	**10** R_1_ = H, R_2_ = R	1.07
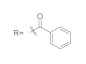	**2** R_1_ = H, R_2_ = R	2.49		**11** R_1_ = R, R_2_ = H	1.33
	**3** R_1_ = R_2_ = R	4.71		**12** R_1_ = R_2_ = R	>10
	**4** R_1_ = R, R_2_ = H	0.91	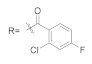	**13** R_1_ = H, R_2_ = R	1.11
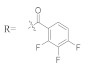	**5** R_1_ = H, R_2_ = R	0.91		**14** R_1_ = R, R_2_ = H	1.76
	**6** R_1_ = R, R_2_ = H	1.22		**15** R_1_ = R_2_ = R	>10
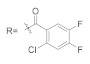	**7** R_1_ = H, R_2_ = R	0.84	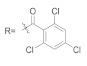	**16** R_1_ = H, R_2_ = R	>10
	**8** R_1_ = R, R_2_ = H	2.06	Doxorubicin		0.06
	**9** R_1_ = R_2_ = R	>10			

Note: ^a^ Results were the average of three independent experiments, each performed in duplicate. Standard deviations were less than ± 10%.

## Data Availability

Data are contained within the article or Supplementary Materials.

## Supplementary Materials

The following are available online at https://www.mdpi.com/article/10.3390/md20010026/s1, Figures S1–S48: ^1^H NMR, ^13^C NMR, and HRESIMS/ESIMS spectra of compounds **1**–**16**.
