# Supplementary material for: Semi-Synthesis, Cytotoxic Evaluation, and Structure—Activity Relationships of Brefeldin A Derivatives with Antileukemia Activity
Source: Mar Drugs. 2021 Dec 24;20(1):26. doi: 10.3390/md20010026 (PMC8777696; doi:10.3390/md20010026)
Supplement: Supplementary file 1 [file marinedrugs-20-00026-s001.zip › marinedrugs-1485592-SI.pdf]

Semi-synthesis, Cytotoxic Evaluation, and Structure-Activity Relationships of Brefeldin A Derivatives with Antileukemia Activity

Xu-Xiu Lu [1,2,#], Yao-Yao Jiang [1,2,#], Yan-Wei Wu [1,2], Guang-Ying Chen [3], Chang-Lun Shao [1,2], Yu-Cheng Gu [4], Ming Liu [1,2,5,*] and Mei-Yan Wei [1,6,*]

[1] Key Laboratory of Marine Drugs, The Ministry of Education of China, School of Medicine and Pharmacy, Ocean University of China, Qingdao 266003, China; 11200811024@stu.ouc.edu.cn (X.-X.L.); jiangyaoyao1226@163.com (Y.-Y.J.); wuyanwei1214@163.com (Y.-W.W.); lmouc@ouc.edu.cn (M.L.); shaochanglun@163.com (C.-L.S.); mywei95@126.com (M.-Y.W.)

[2] Laboratory for Marine Drugs and Bioproducts, Qingdao National Laboratory for Marine Science and Technology, Qingdao 266200, China

[3] Key Laboratory of Tropical Medicinal Resource Chemistry of Ministry of Education, College of Chemistry and Chemical Engineering, Hainan Normal University, Haikou 571158, China; chgying123@163.com (G.-Y.C.)

[4] Syngenta Jealott's Hill International Research Centre, Bracknell, Berkshire, RG42 6EY, United Kingdom; yucheng.gu@syngenta.com (Y.-C.G.)

[5] State Key Laboratory for Chemistry and Molecular Engineering of Medicinal Resources, Guangxi Normal University, Guilin 541001, China

[6] College of Food Science and Engineering, Ocean University of China, Qingdao 266003, China

* Correspondence: lmouc@ouc.edu.cn (M.L.); mywei95@126.com (M.-Y.W.); Tel.: +86-532-8203-1980 (M.L.); +86-532-8203-1381 (M.-Y.W.)

[#] These authors contributed equally to this work.

Content of Supporting Information

Compound Characterization Spectra

Figure S1. ^{1}H NMR (500 MHz, DMSO-d_6) spectrum of compound **1**.

Figure S2. ^{13}C NMR (125 MHz, DMSO-d_6) spectrum of compound **1**.

Figure S3. ESIMS spectrum of compound **1.**

Figure S4. ^{1}H NMR (400 MHz, $CDCl_3$) spectrum of compound **2**.

Figure S5. ^{13}C NMR (100 MHz, $CDCl_3$) spectrum of compound **2**.

Figure S6. ESIMS spectrum of compound **2.**

Figure S7. ^{1}H NMR (500 MHz, $CDCl_3$) spectrum of compound **3**.

Figure S8. ^{13}C NMR (100 MHz, $CDCl_3$) spectrum of compound **3**.

Figure S9. HRESIMS spectrum of compound **3.**

Figure S10. ^{1}H NMR (400 MHz, $CDCl_3$) spectrum of compound **4**.

Figure S11. ^{13}C NMR (100 MHz, $CDCl_3$) spectrum of compound **4**.

Figure S12. HRESIMS spectrum of compound **4**.

Figure S13. ^{1}H NMR (500 MHz, $CDCl_3$) spectrum of compound **5**.

Figure S14. ^{13}C NMR (125 MHz, $CDCl_3$) spectrum of compound **5**.

Figure S15. HRESIMS spectrum of compound **5**.

Figure S16. ^{1}H NMR (500 MHz, $CDCl_3$) spectrum of compound **6**.

Figure S17. ^{13}C NMR (125 MHz, $CDCl_3$) spectrum of compound **6**.

Figure S18. HRESIMS spectrum of compound **6**.

Figure S19. ^{1}H NMR (400 MHz, $CDCl_3$) spectrum of compound **7**.

Figure S20. ^{13}C NMR (100 MHz, $CDCl_3$) spectrum of compound **7**.

Figure S21. HRESIMS spectrum of compound **7**.

Figure S22. ^{1}H NMR (400 MHz, $CDCl_3$) spectrum of compound **8**.

Figure S23. ^{13}C NMR (100 MHz, $CDCl_3$) spectrum of compound **8**.

Figure S24. HRESIMS spectrum of compound **8**.

Figure S25. ^{1}H NMR (400 MHz, $CDCl_3$) spectrum of compound **9**.

Figure S26. ^{13}C NMR (100 MHz, $CDCl_3$) spectrum of compound **9**.

Figure S27. HRESIMS spectrum of compound **9**.

Figure S28. ^{1}H NMR (600 MHz, $CDCl_3$) spectrum of compound **10**.

Figure S29. ^{13}C NMR (150 MHz, $CDCl_3$) spectrum of compound **10**.

Figure S30. HRESIMS spectrum of compound **10**.

Figure S31. ^{1}H NMR (600 MHz, $CDCl_3$) spectrum of compound **11.**

Figure S32. ^{13}C NMR (150 MHz, $CDCl_3$) spectrum of compound **11.**

Figure S33. HRESIMS spectrum of compound **11**.

Figure S34. ^{1}H NMR (600 MHz, $CDCl_3$) spectrum of compound **12.**

Figure S35. ^{13}C NMR (125 MHz, $CDCl_3$) spectrum of compound **12.**

Figure S36. HRESIMS spectrum of compound **12**.

Figure S37. ^{1}H NMR (400 MHz, $CDCl_3$) spectrum of compound **13.**

Figure S38. ^{13}C NMR (100 MHz, $CDCl_3$) spectrum of compound **13.**

Figure S39. HRESIMS spectrum of compound **13**.

Figure S40. ^{1}H NMR (600 MHz, $CDCl_3$) spectrum of compound **14.**

Figure S41. ^{13}C NMR (150 MHz, $CDCl_3$) spectrum of compound **14.**

Figure S42. HRESIMS spectrum of compound **14**.

Figure S43. ^{1}H NMR (600 MHz, $CDCl_3$) spectrum of compound **15.**

Figure S44. ^{13}C NMR (150 MHz, $CDCl_3$) spectrum of compound **15.**

Figure S45. HRESIMS spectrum of compound **15**.

Figure S46. ^{1}H NMR (500 MHz, $CDCl_3$) spectrum of compound **16.**

Figure S47. ^{13}C NMR (100 MHz, $CDCl_3$) spectrum of compound **16.**

Figure S48. HRESIMS spectrum of compound **16**.

*Brefeldin A (**1**)*

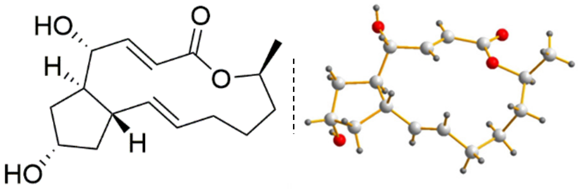

Known compound, white, amorphous powder; ^{1}H NMR (500 MHz, DMSO-d_6) δ 7.34 (1H, dd, J = 15.5, 3.0 Hz, H-3), 5.75–5.60 (2H, overlapped, H-2, H-11), 5.20 (1H, dd, J = 15.2, 9.6 Hz, H-10), 5.10 (1H, s, 4-OH), 4.71 (1H, m, H-15), 4.48 (1H, s, 7-OH), 4.04 (1H, m, H-7), 3.92 (1H, d, J = 9.2 Hz, H-4), 2.30 (1H, m, H-9), 2.02–1.87 (2H, overlapped, H-8, H-12), 1.87–1.60 (6H, overlapped, H-5, H-6a, H-6b, H-12, H-13, H-14), 1.47 (1H, m, H-14), 1.30 (1H, m, H-8), 1.18 (3H, d, J = 6.3 Hz,16-CH_3), 0.75 (1H, m, H-13); ^{13}C NMR (125 MHz, DMSO-d_6) δ 165.7 (C-1), 154.4 (C-3), 137.1 (C-10), 129.2 (C-11), 116.3 (C-2), 74.3 (C-4), 70.9 (C-15), 70.5 (C-7), 51.7 (C-5), 43.3 (C-9), 43.1 (C-8), 40.9 (C-6), 33.4 (C-14), 31.5 (C-12), 26.5 (C-13), 20.7 (C-16); ESIMS *m/z* 281.17 $[M + H]^+$, 263.19 $[M + H - H_2O]^+$, 245.19 $[M + H - 2H_2O]^+$.

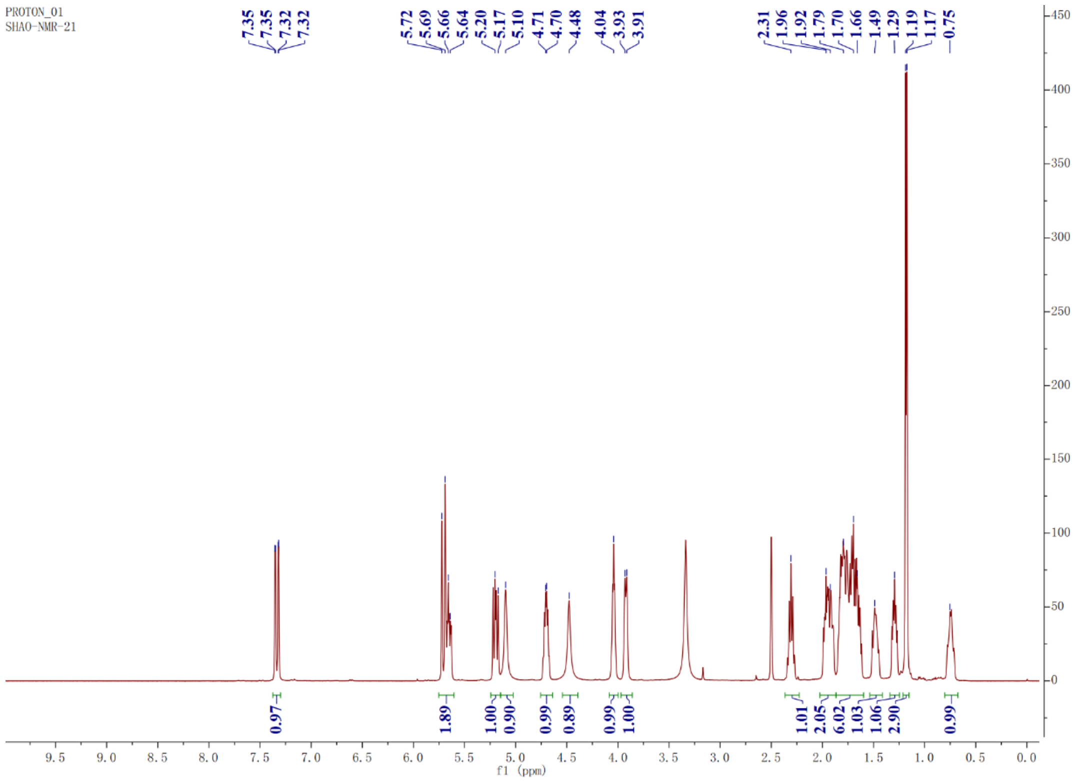

Figure S1. ^{1}H NMR (500 MHz, DMSO-d_6) spectrum of compound **1.**

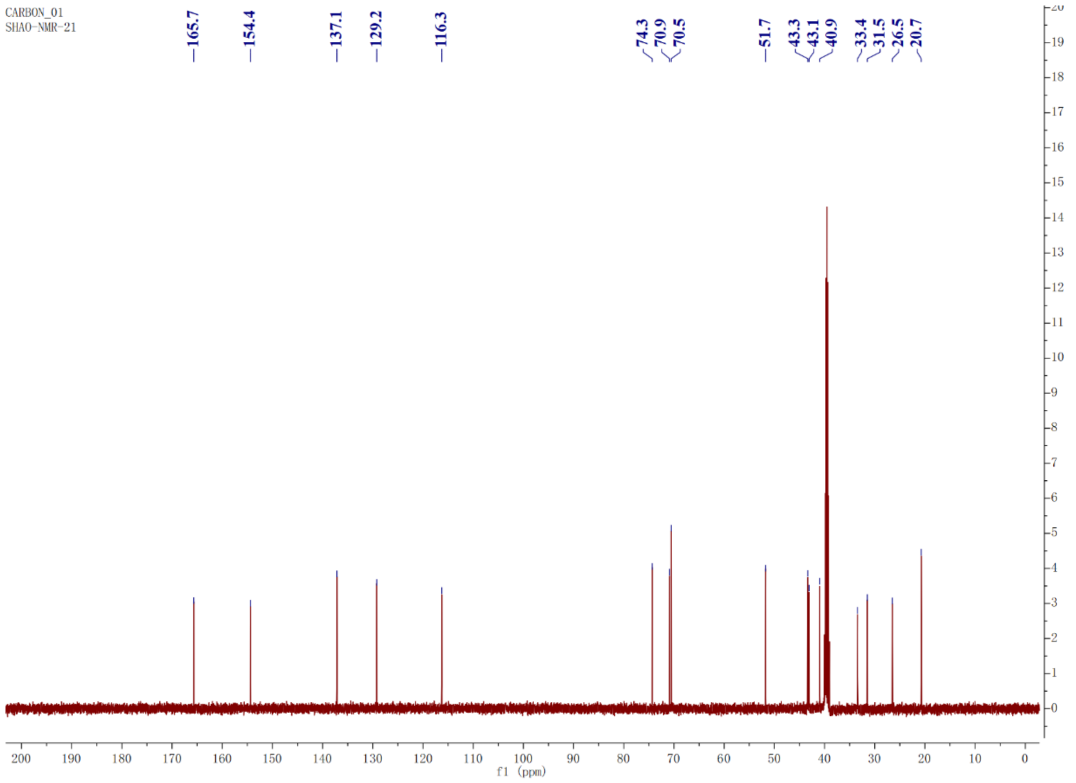

Figure S2. ^{13}C NMR (125 MHz, DMSO-d_6) spectrum of compound **1.**

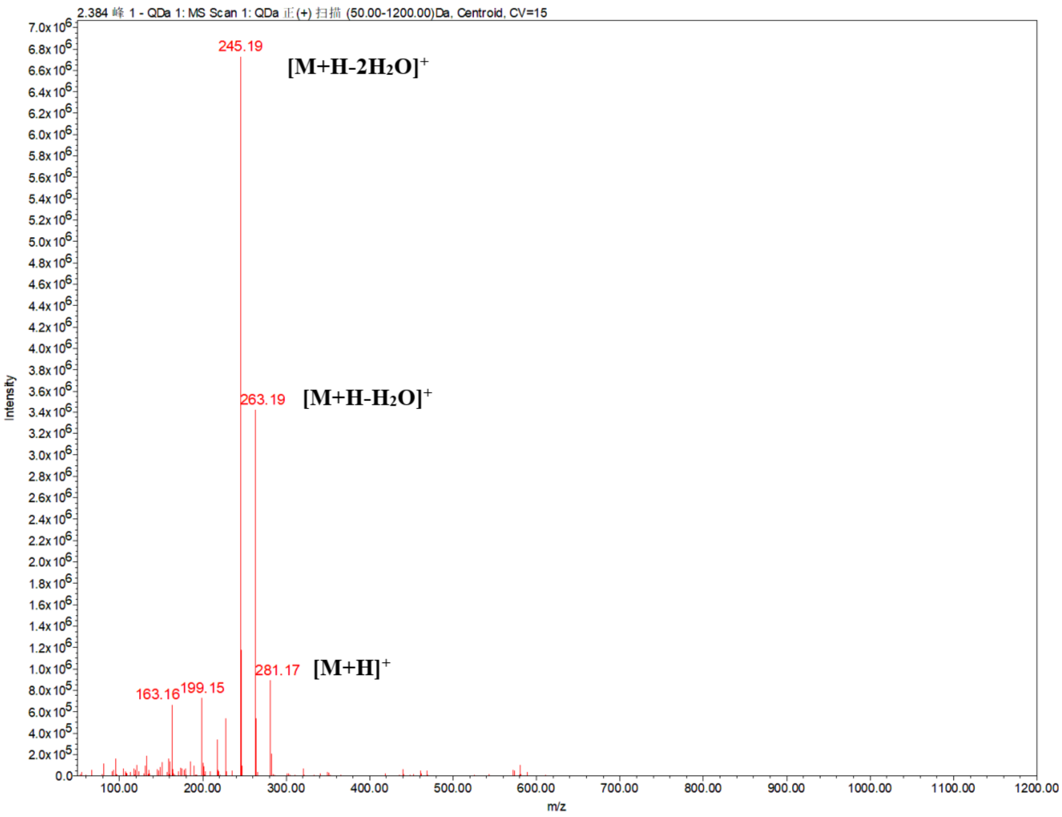

Figure S3. ESIMS spectrum of compound **1.**

*Brefeldin A 7-O-benzoate (**2**)*

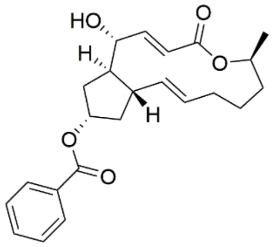

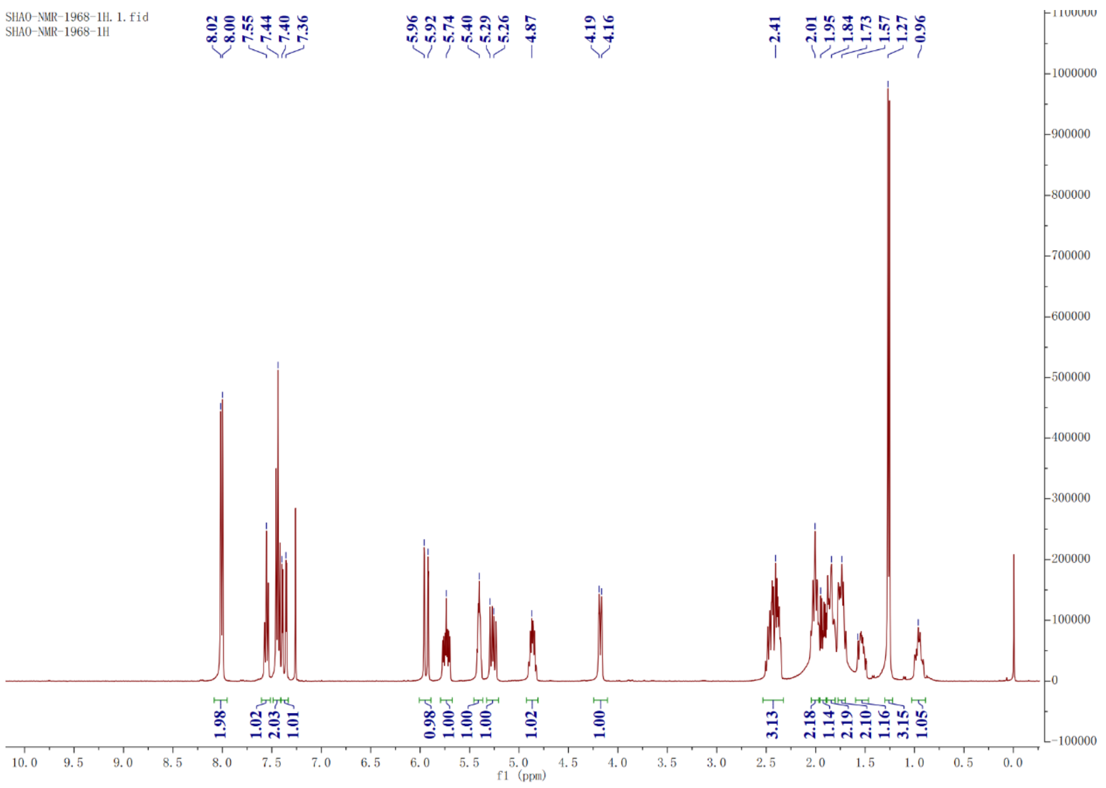

Figure S4. ^{1}H NMR (400 MHz, $CDCl_3$) spectrum of compound **2.**

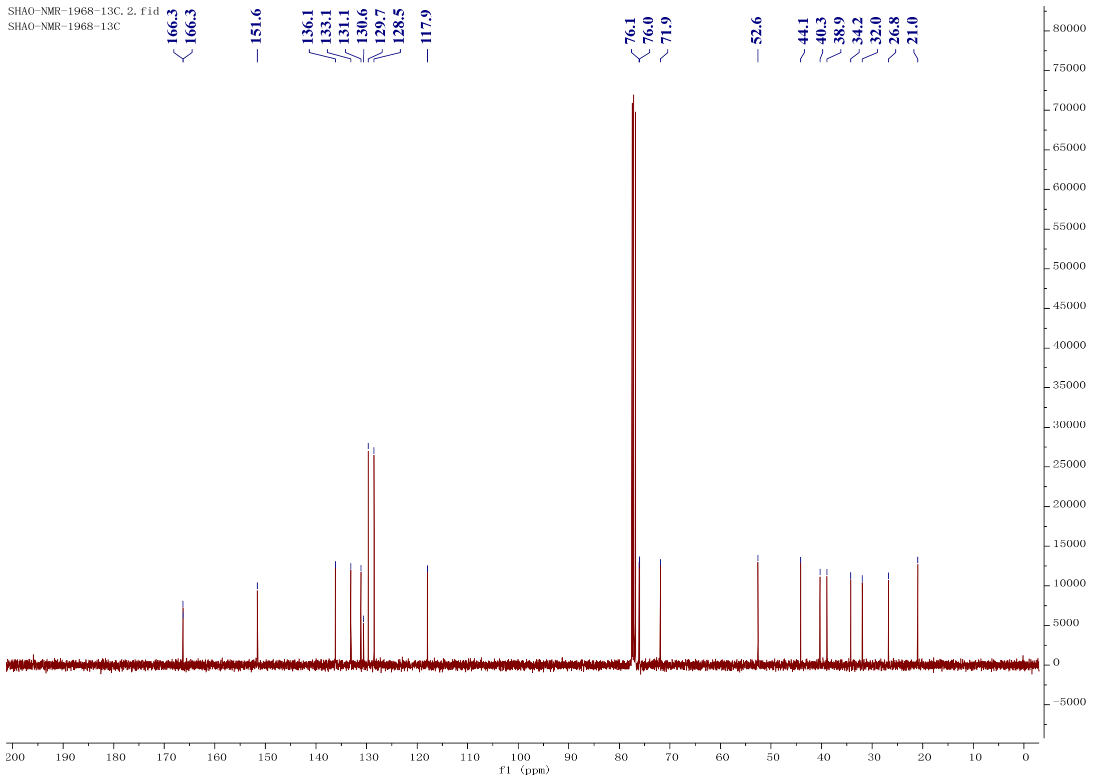

Figure S5. ^{13}C NMR (100 MHz, $CDCl_3$) spectrum of compound **2.**

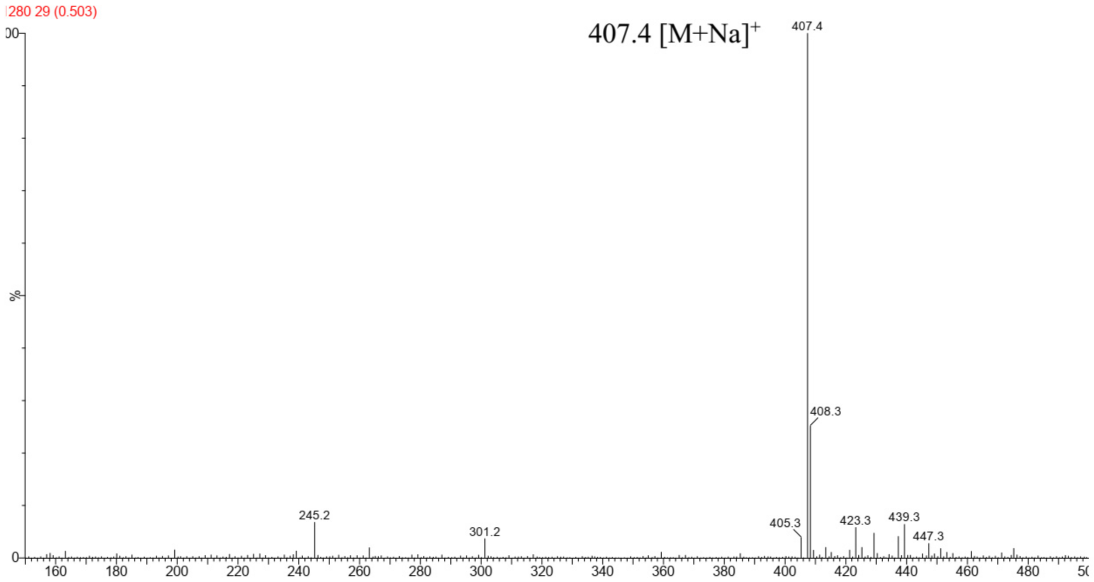

Figure S6. ESIMS spectrum of compound **2.**

*Brefeldin A 4,7-O-dibenzoate (**3**)*

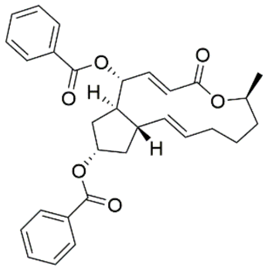

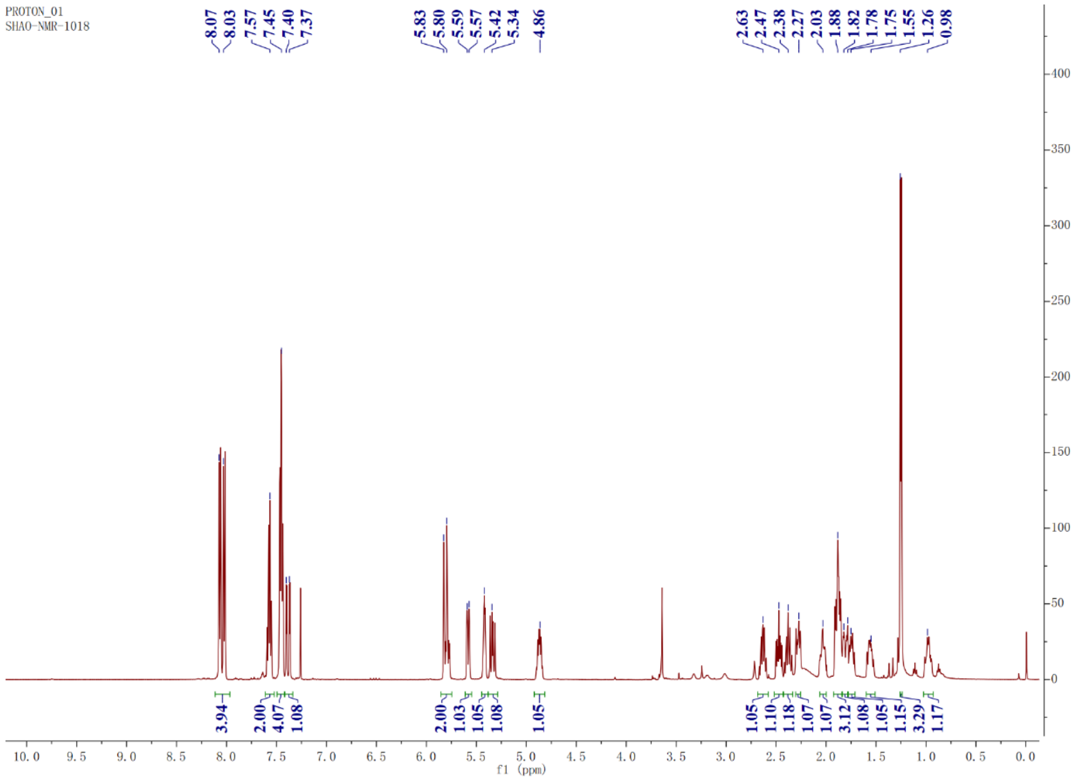

Figure S7. ^{1}H NMR (500 MHz, $CDCl_3$) spectrum of compound **3.**

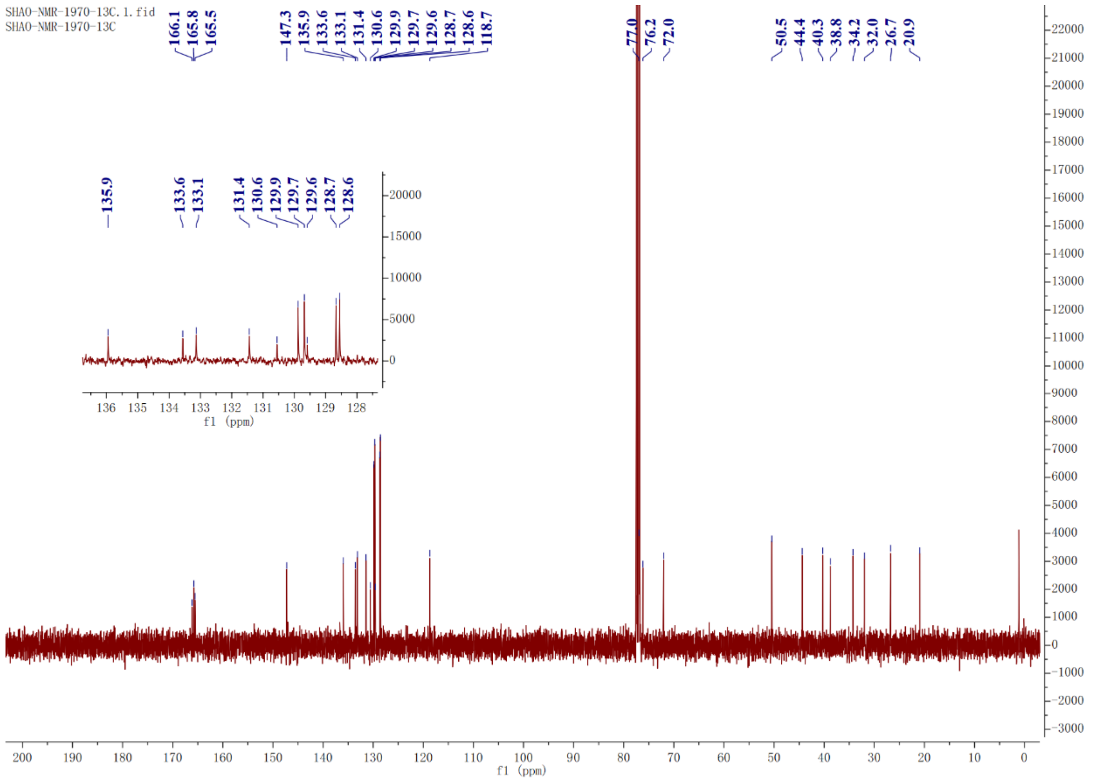

Figure S8. ^{13}C NMR (100 MHz, $CDCl_3$) spectrum of compound **3.**

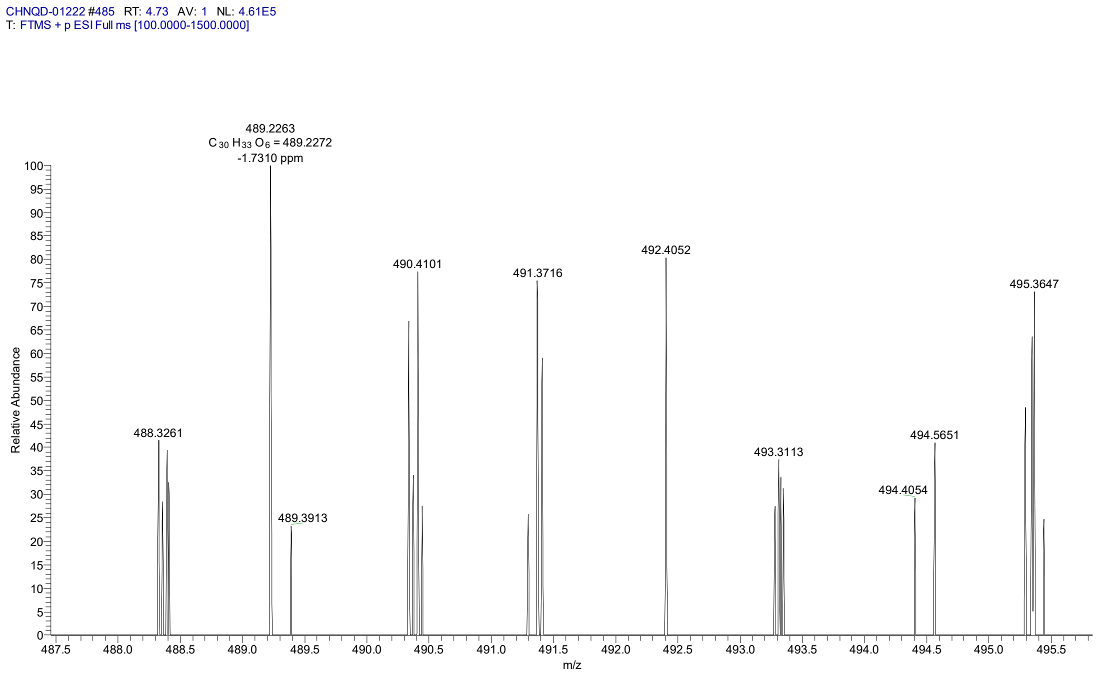

Figure S9. ESIMS spectrum of compound **3.**

*Brefeldin A 4-O-benzoate (**4**)*

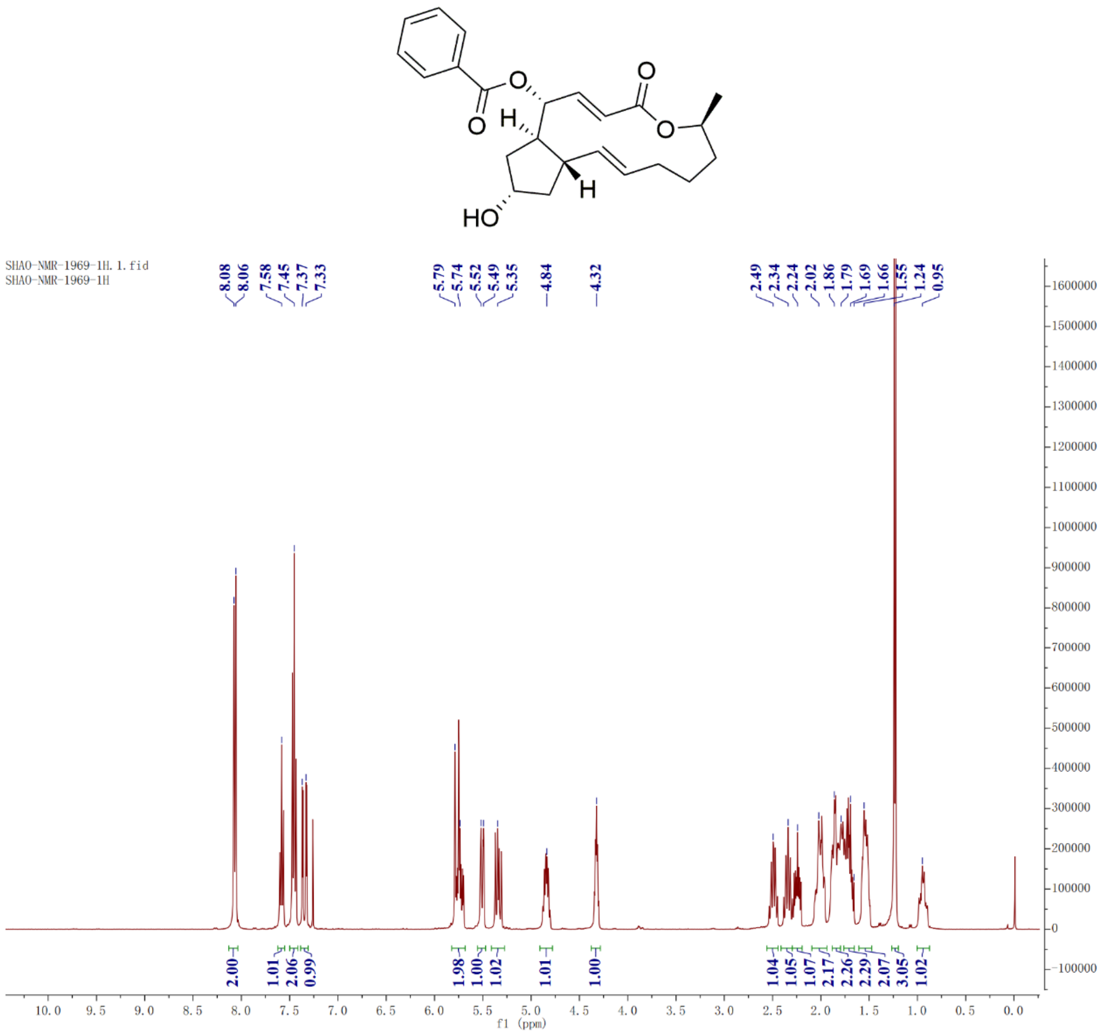

Figure S10. ^1H NMR (400 MHz, $CDCl_3$) spectrum of compound **4.**

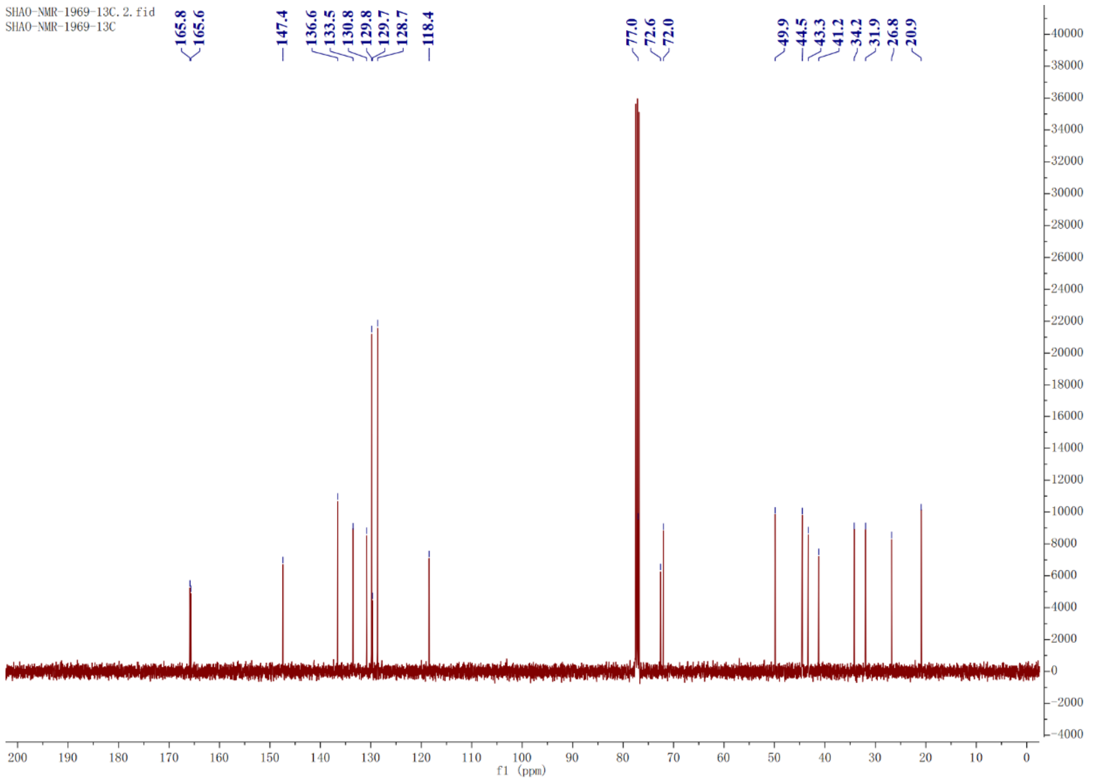

Figure S11. ^{13}C NMR (100 MHz, $CDCl_3$) spectrum of compound **4.**

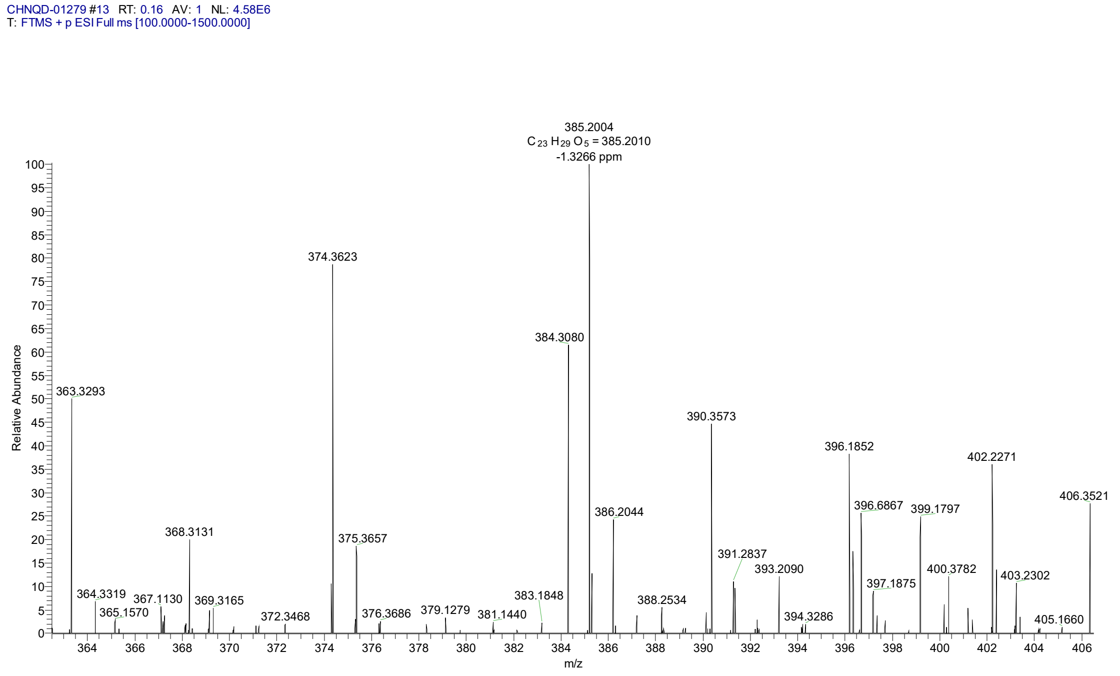

Figure S12. HRESIMS spectrum of compound **4**.

*Brefeldin A 7-O-(2,3,4)-trifluorobenzoate (**5**)*

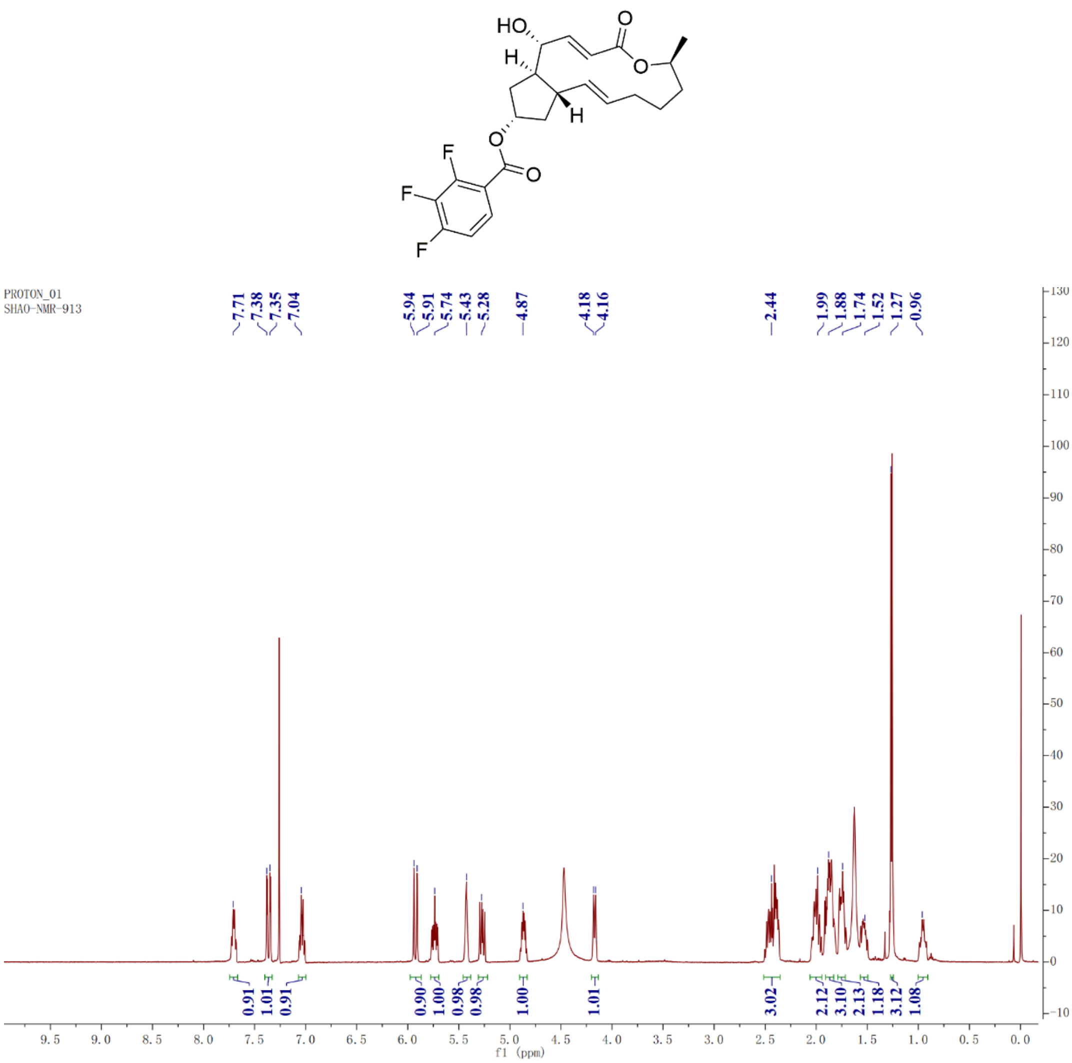

Figure S13. ^{1}H NMR (500 MHz, $CDCl_3$) spectrum of compound **5.**

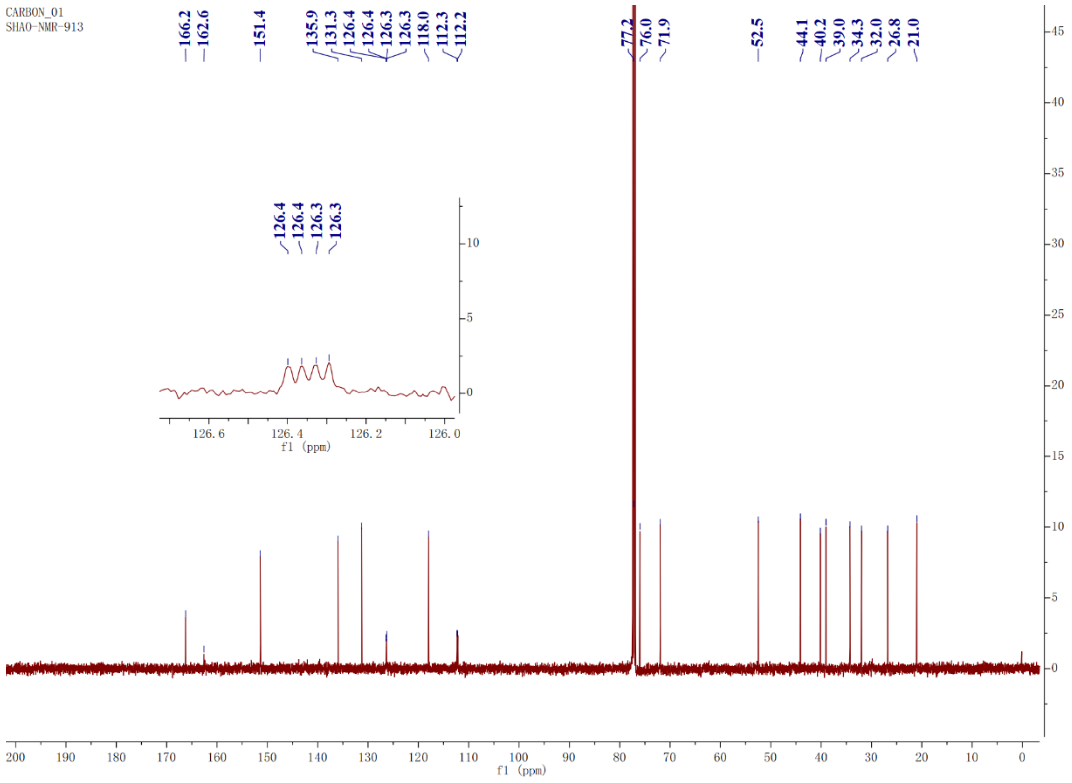

Figure S14. ^{13}C NMR (125 MHz, $CDCl_3$) spectrum of compound **5.**

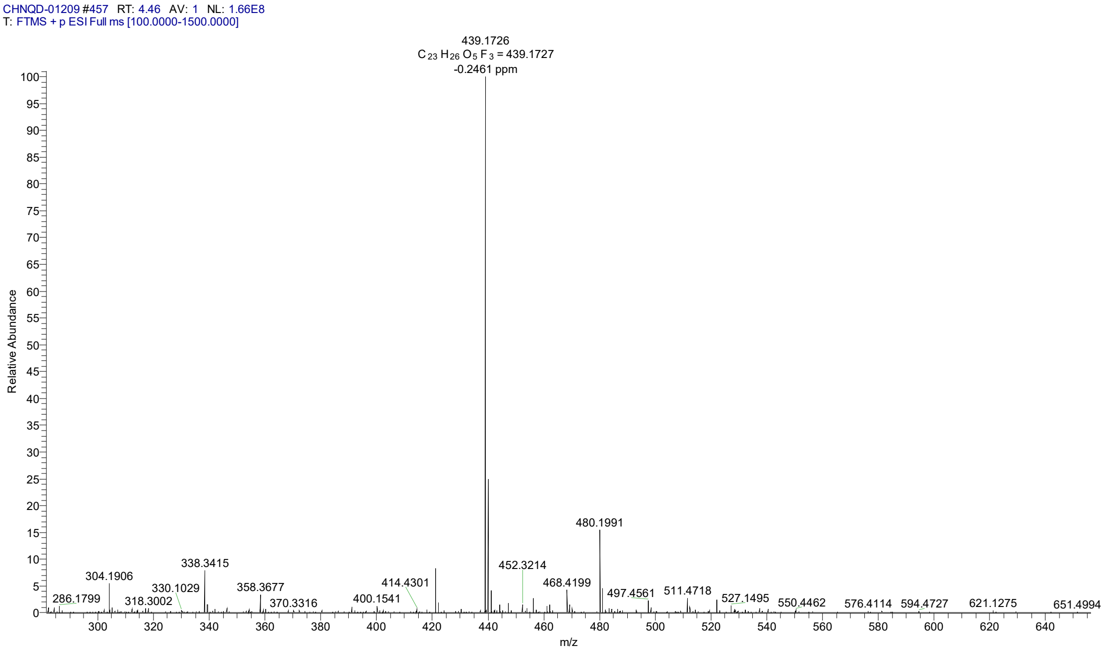

Figure S15. HRESIMS spectrum of compound **5**.

*Brefeldin A 4-O-(2,3,4)-trifluorobenzoate (**6**)*

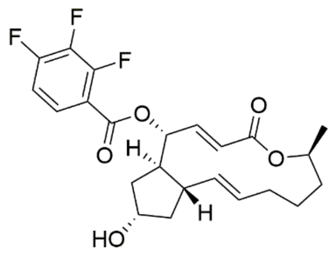

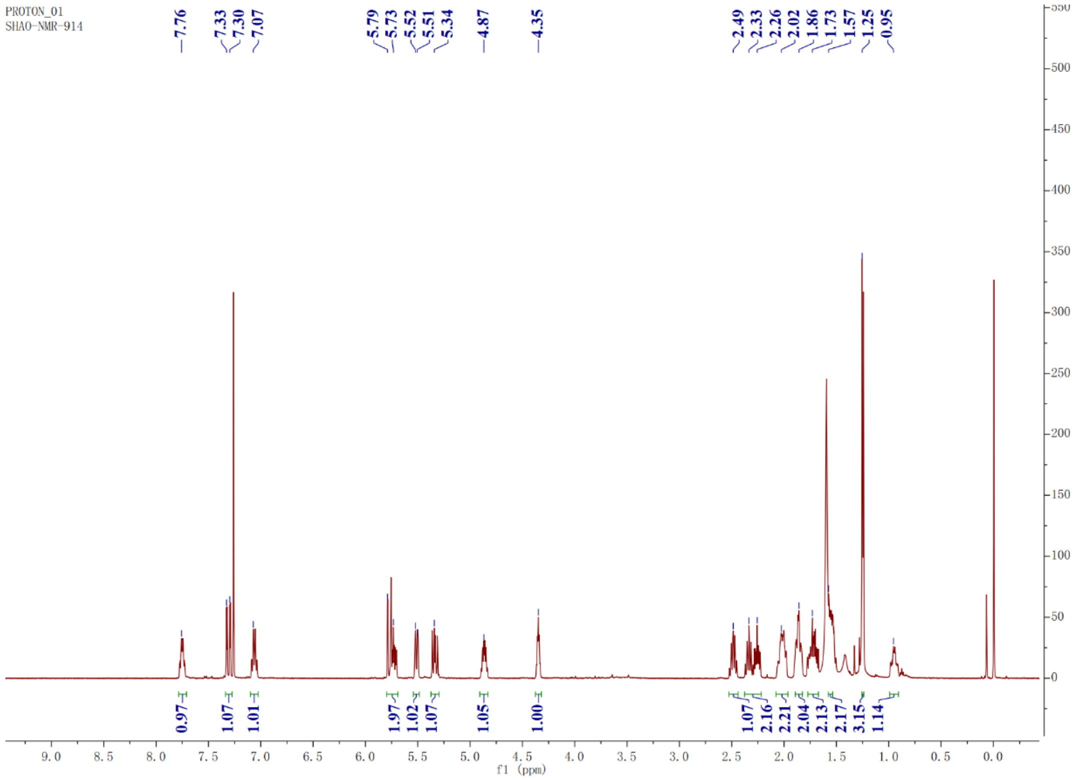

Figure S16. ^{1}H NMR (500 MHz, $CDCl_3$) spectrum of compound **6.**

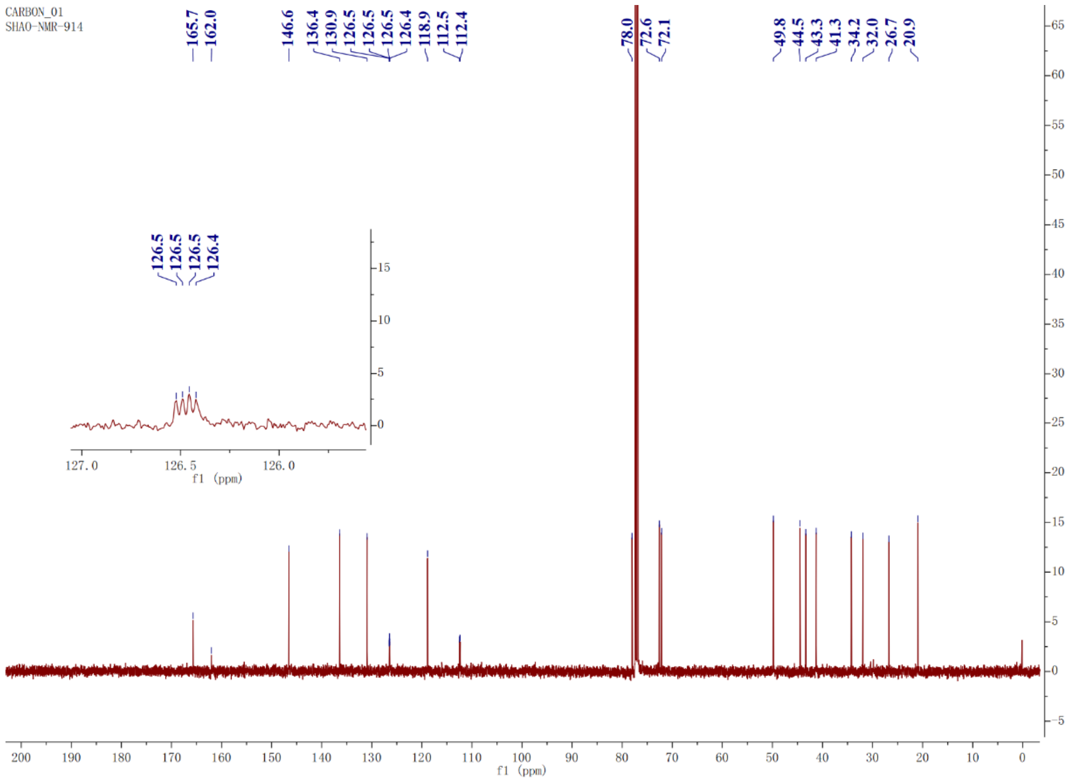

Figure S17. ^{13}C NMR (125 MHz, $CDCl_3$) spectrum of compound **6.**

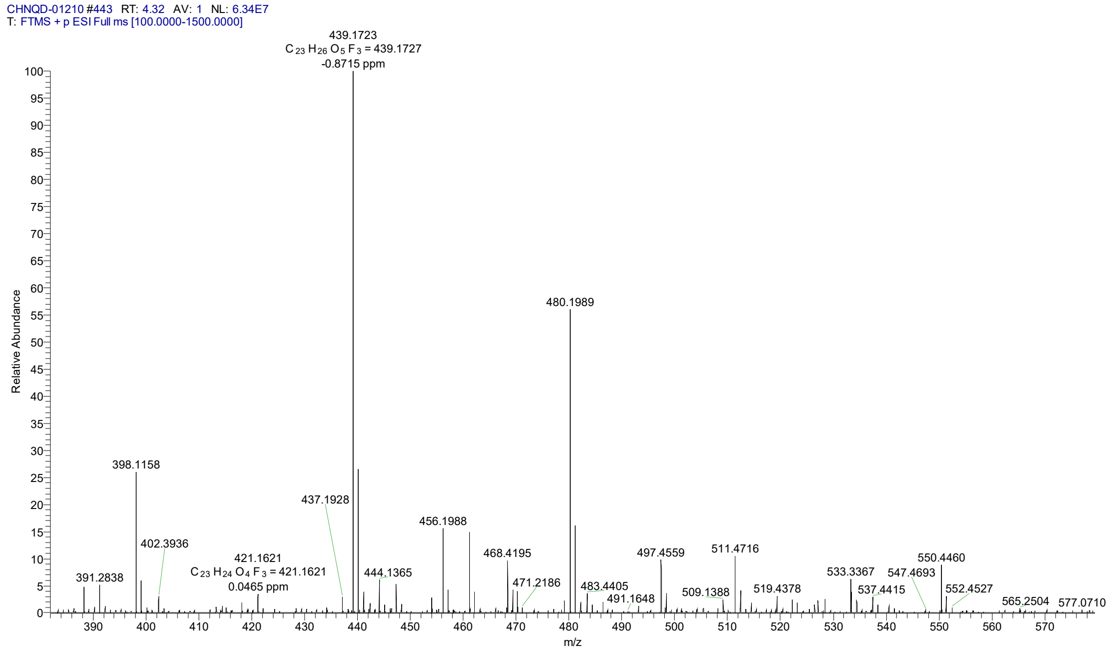

Figure S18. HRESIMS spectrum of compound **6**.

*Brefeldin A 7-O-2-chloro-4,5-difluorobenzoate (**7**)*

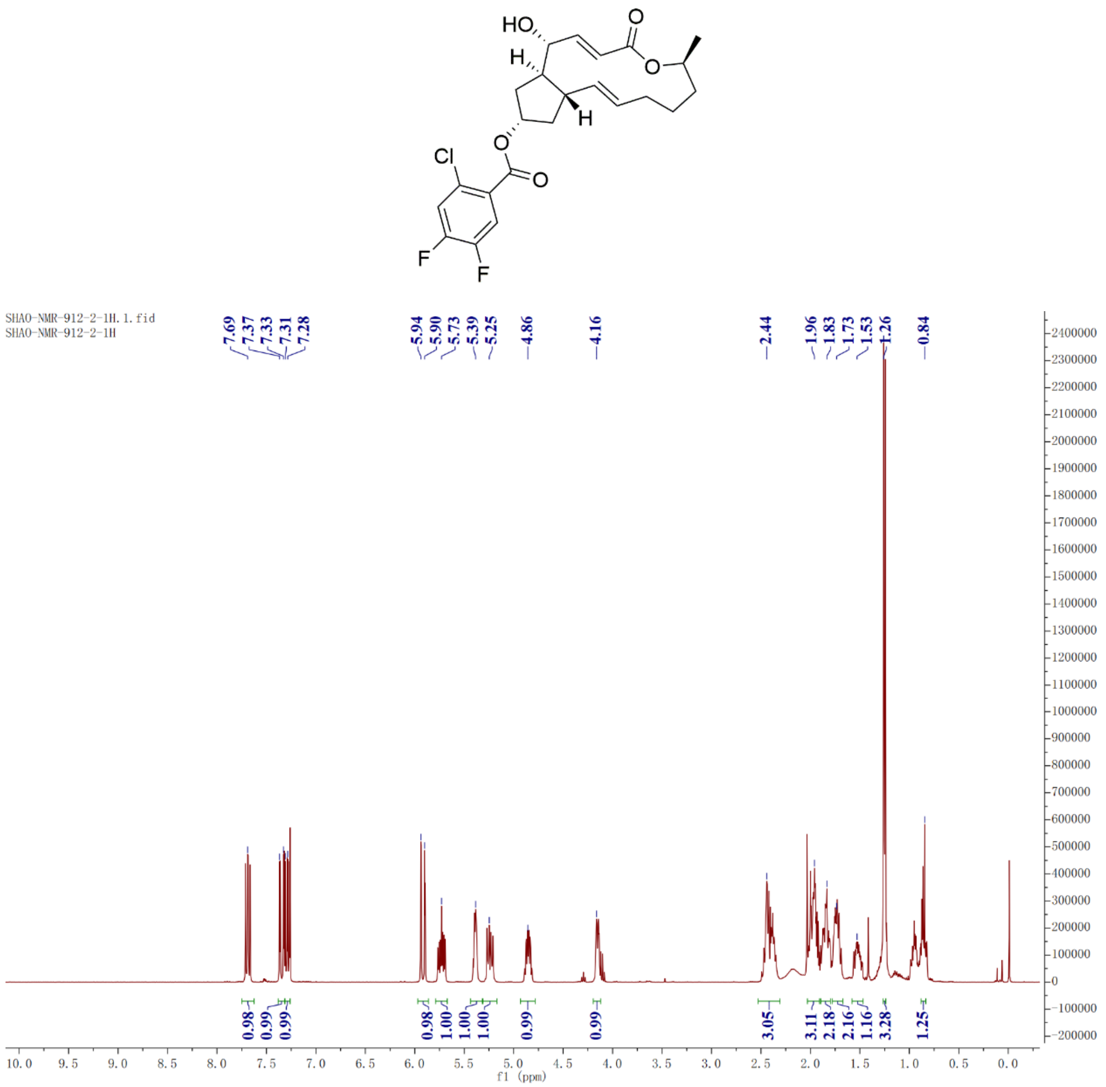

Figure S19. ^{1}H NMR (400 MHz, $CDCl_3$) spectrum of compound **7.**

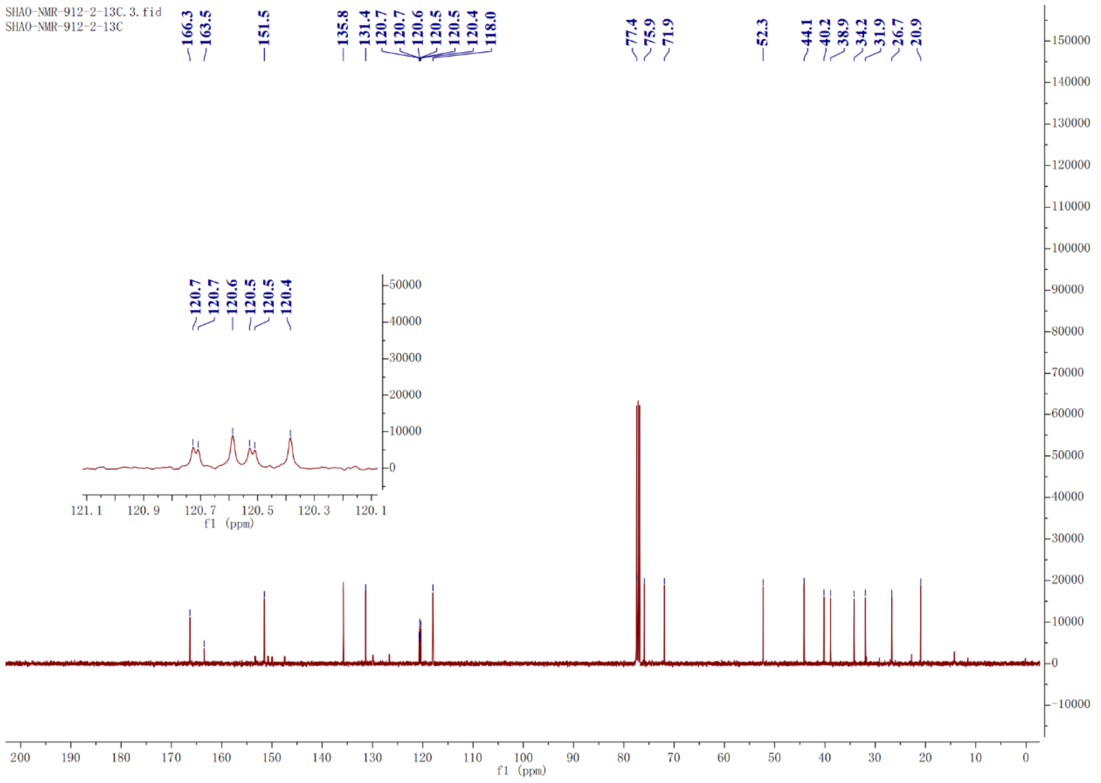

Figure S20. ^{13}C NMR (100 MHz, $CDCl_3$) spectrum of compound **7.**

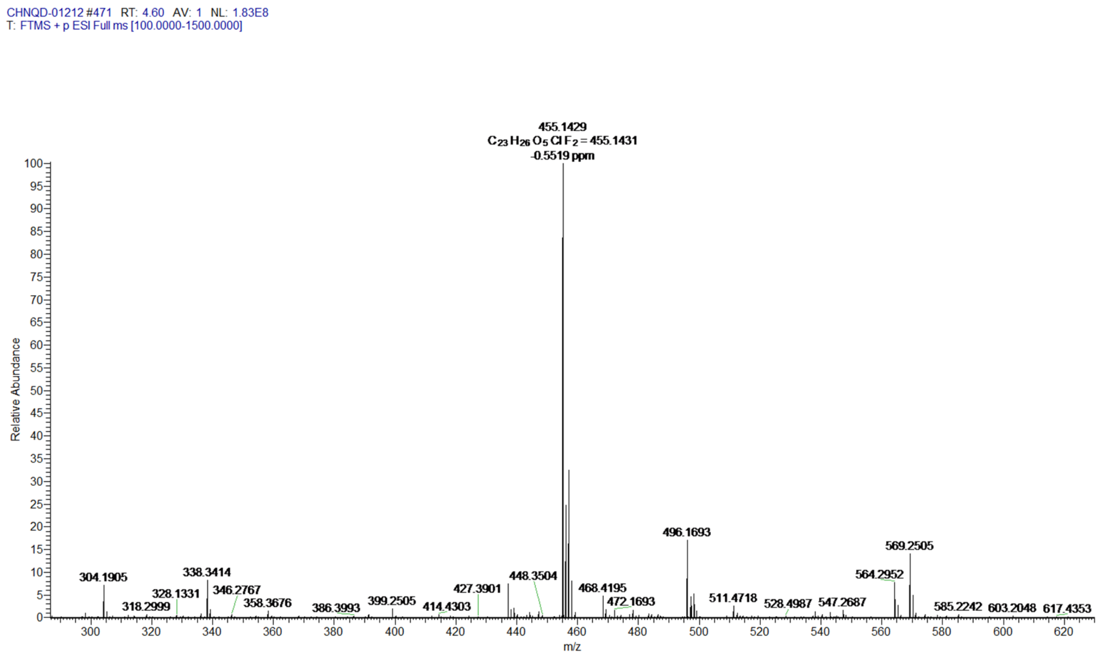

Figure S21. HRESIMS spectrum of compound **7**.

*Brefeldin A 4-O-2-chloro-4,5-difluorobenzoate (**8**)*

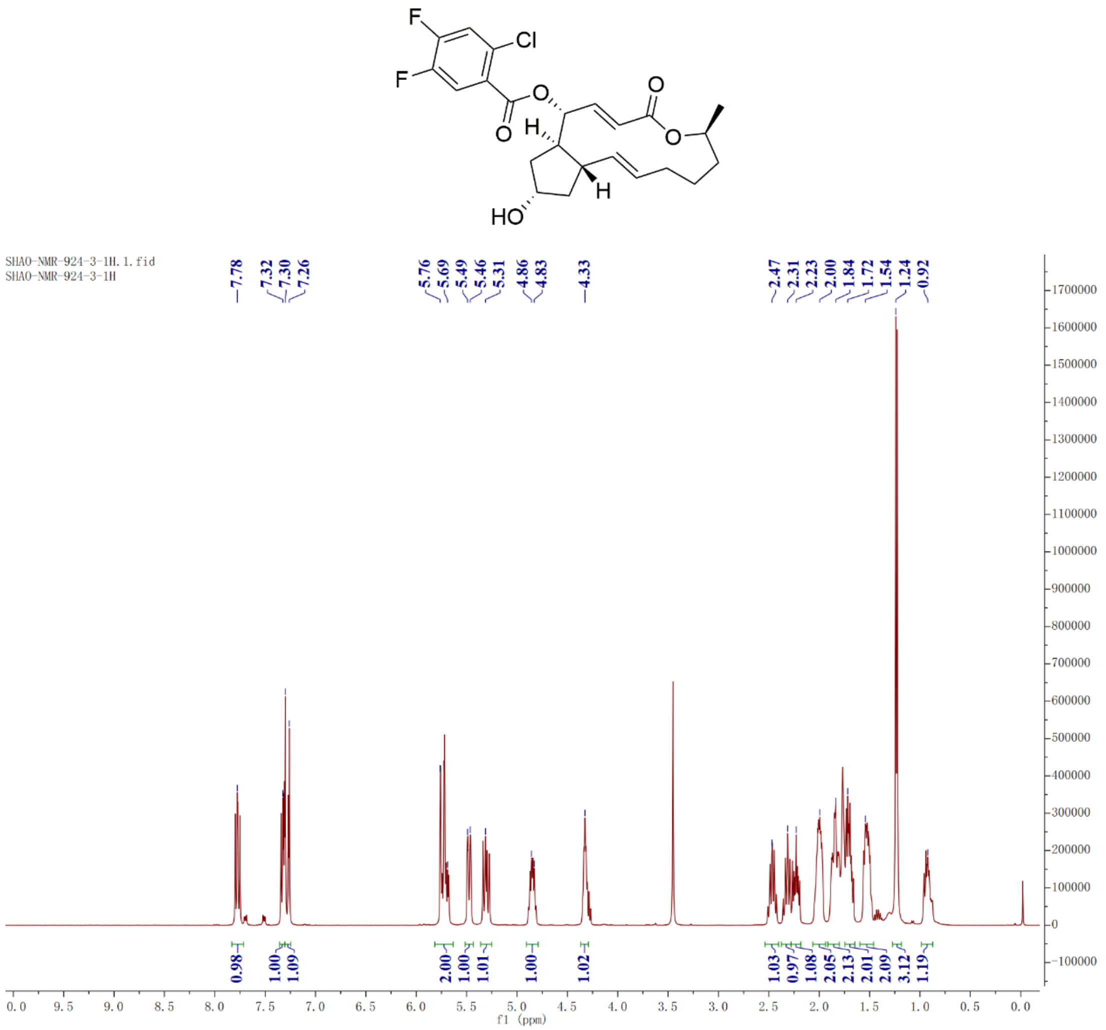

Figure S22. ^{1}H NMR (400 MHz, $CDCl_3$) spectrum of compound **8.**

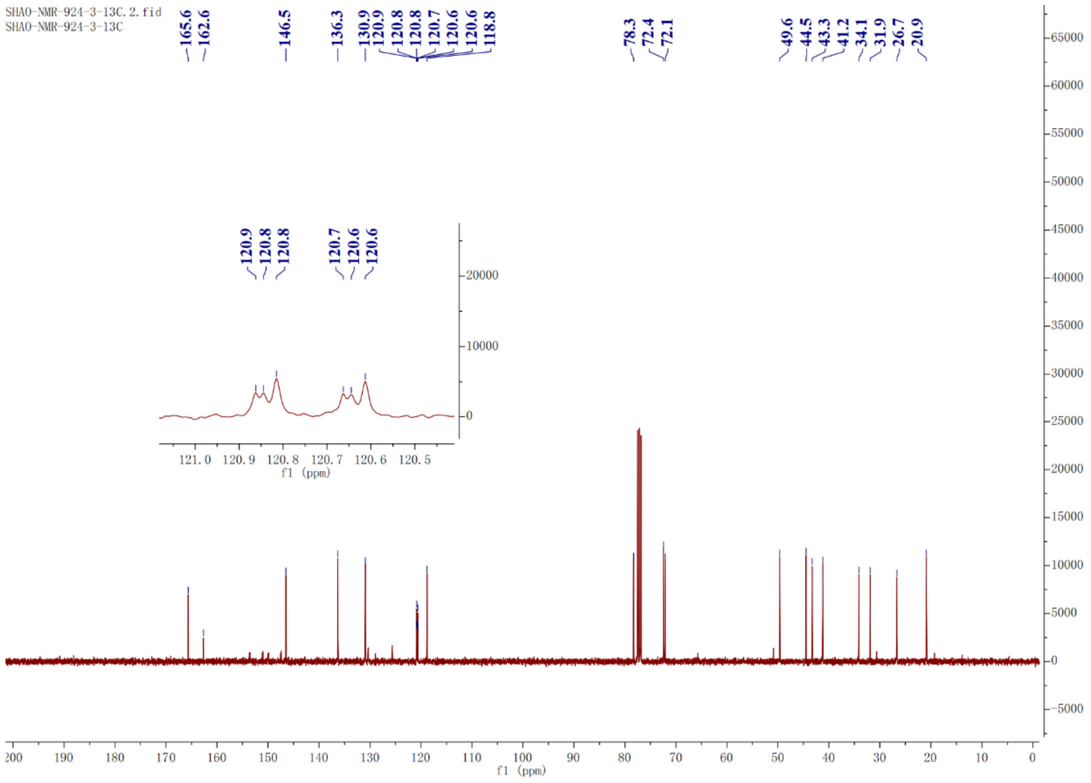

Figure S23. ^{13}C NMR (100 MHz, $CDCl_3$) spectrum of compound **8.**

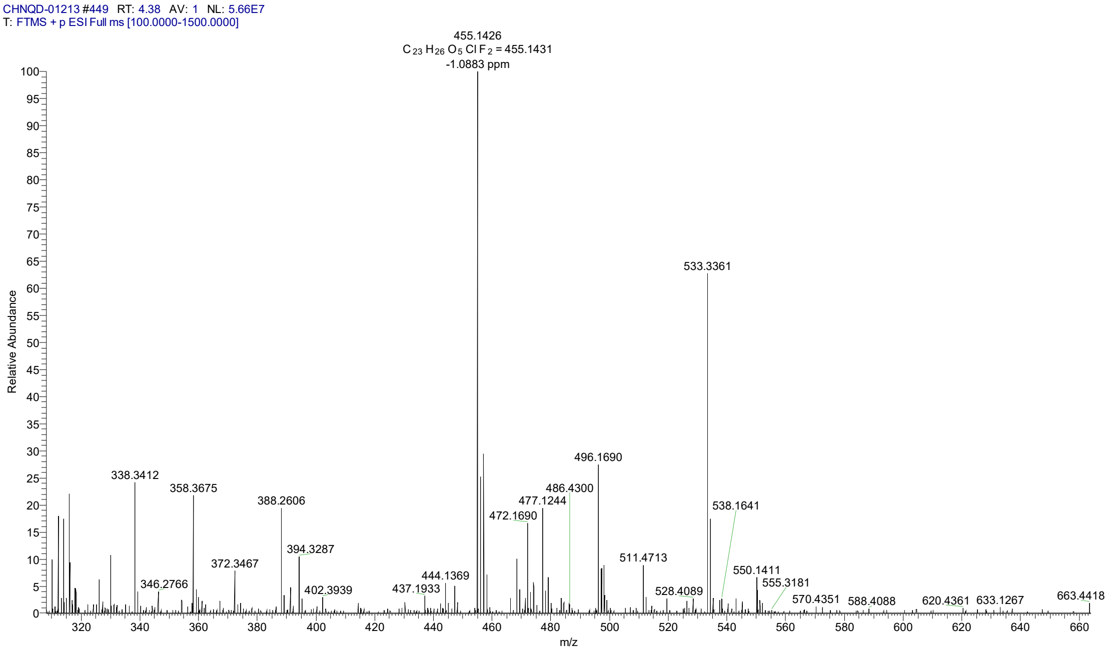

Figure S24. HRESIMS spectrum of compound **8**.

*Brefeldin A 4,7-O-di-2-chloro-4,5-difluorobenzoate (**9**)*

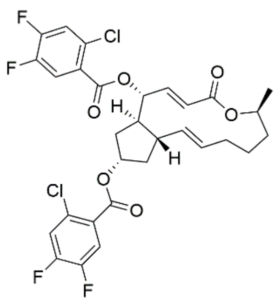

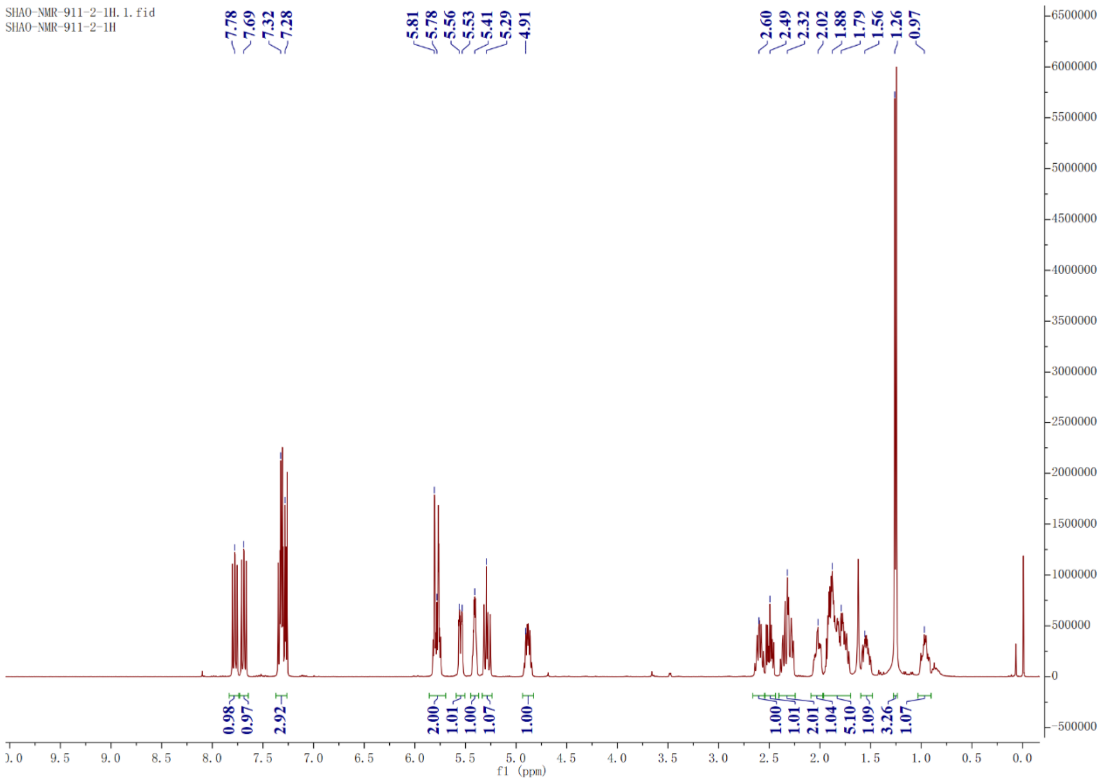

Figure S25. ^{1}H NMR (400 MHz, $CDCl_3$) spectrum of compound **9.**

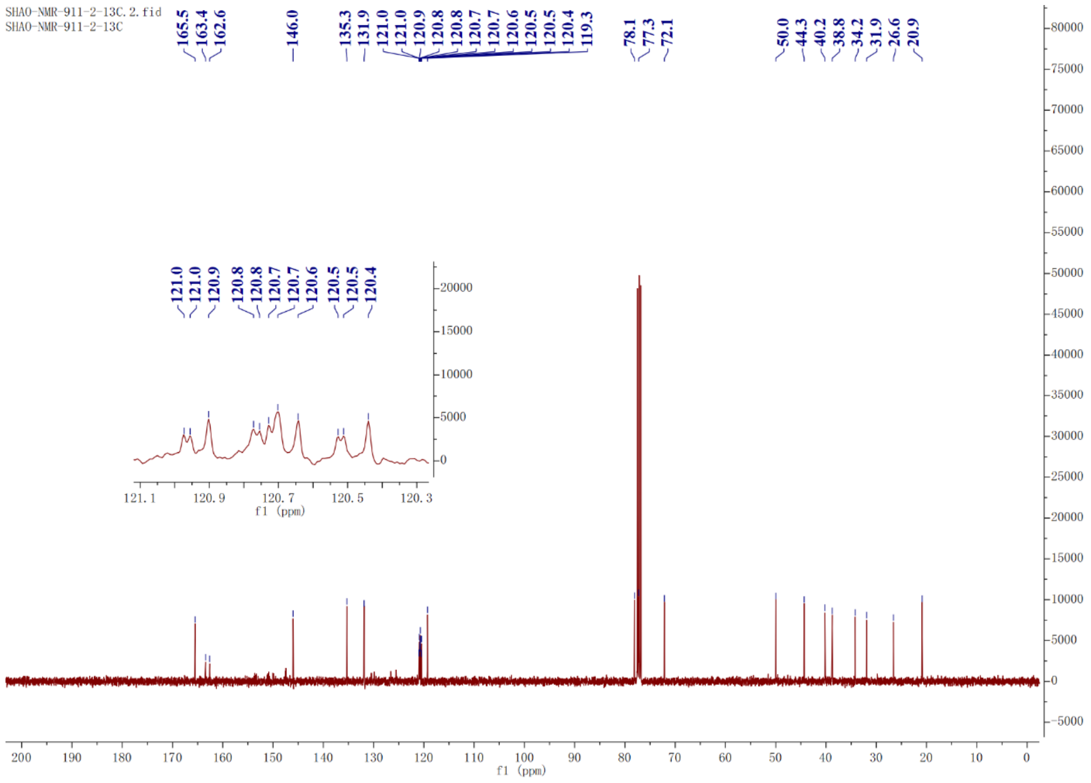

Figure S26. ^{13}C NMR (100 MHz, $CDCl_3$) spectrum of compound **9.**

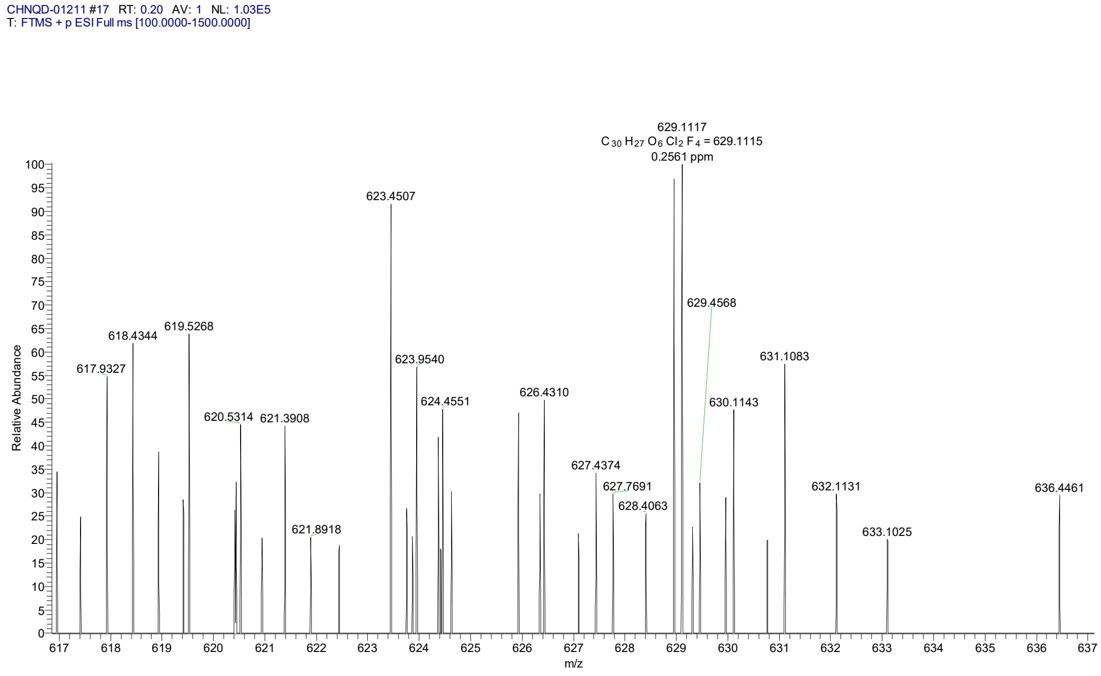

Figure S27. HRESIMS spectrum of compound **9**.

*Brefeldin A 7-O-(4)-chlorobenzoate (**10**)*

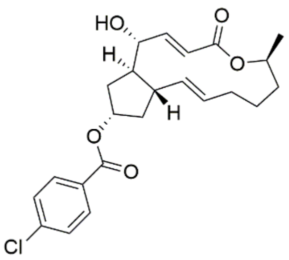

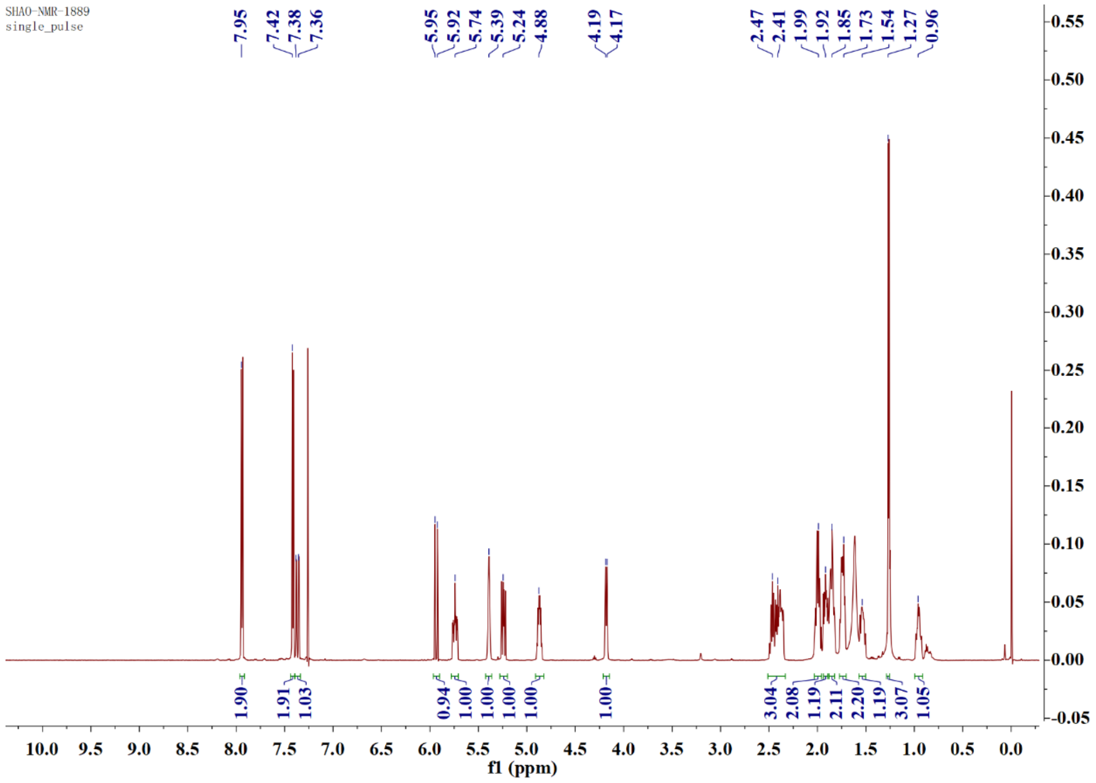

Figure S28. ^{1}H NMR (600 MHz, $CDCl_3$) spectrum of compound **10.**

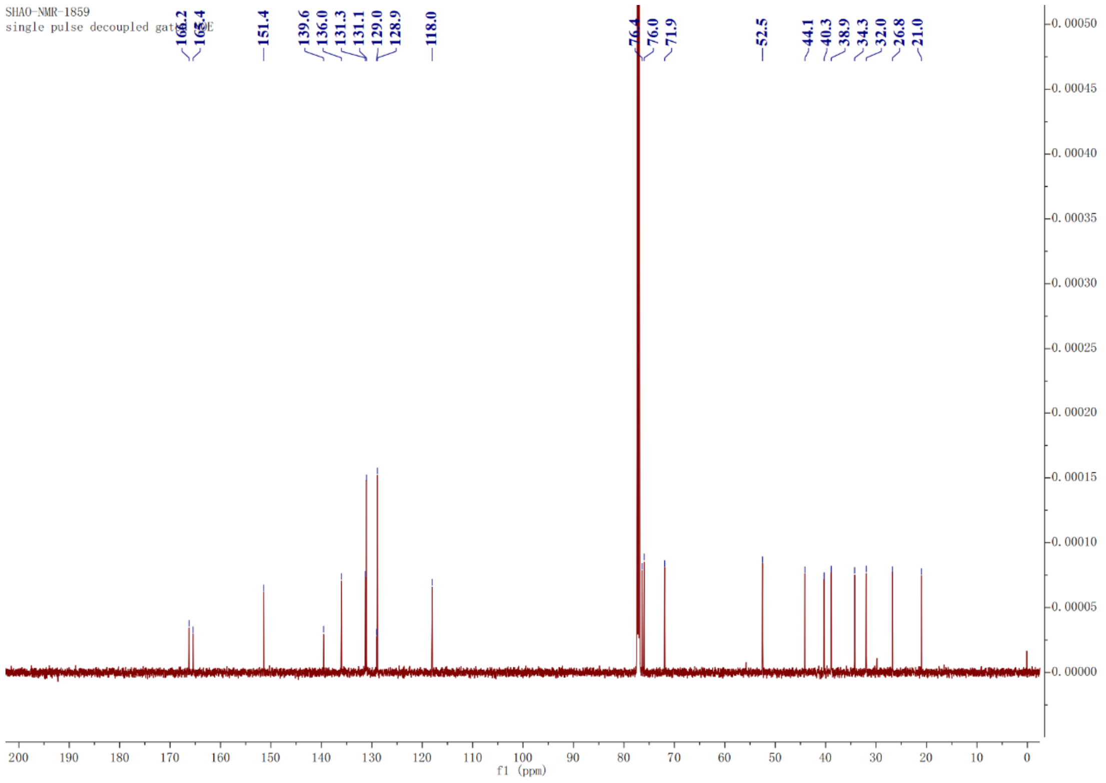

Figure S29. ^{13}C NMR (150 MHz, $CDCl_3$) spectrum of compound **10.**

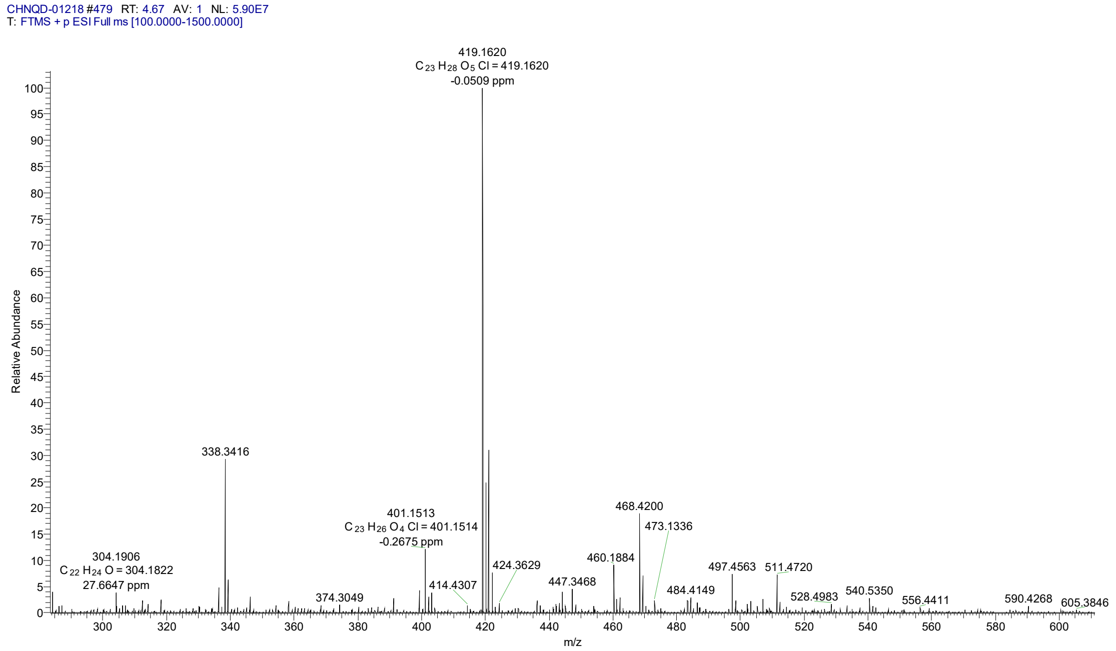

Figure S30. HRESIMS spectrum of compound **10**.

*Brefeldin A 4-O-(4)-chlorobenzoate (**11**)*

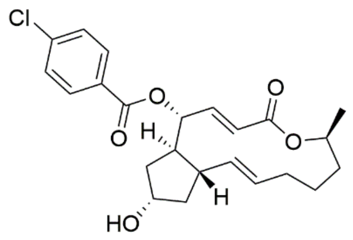

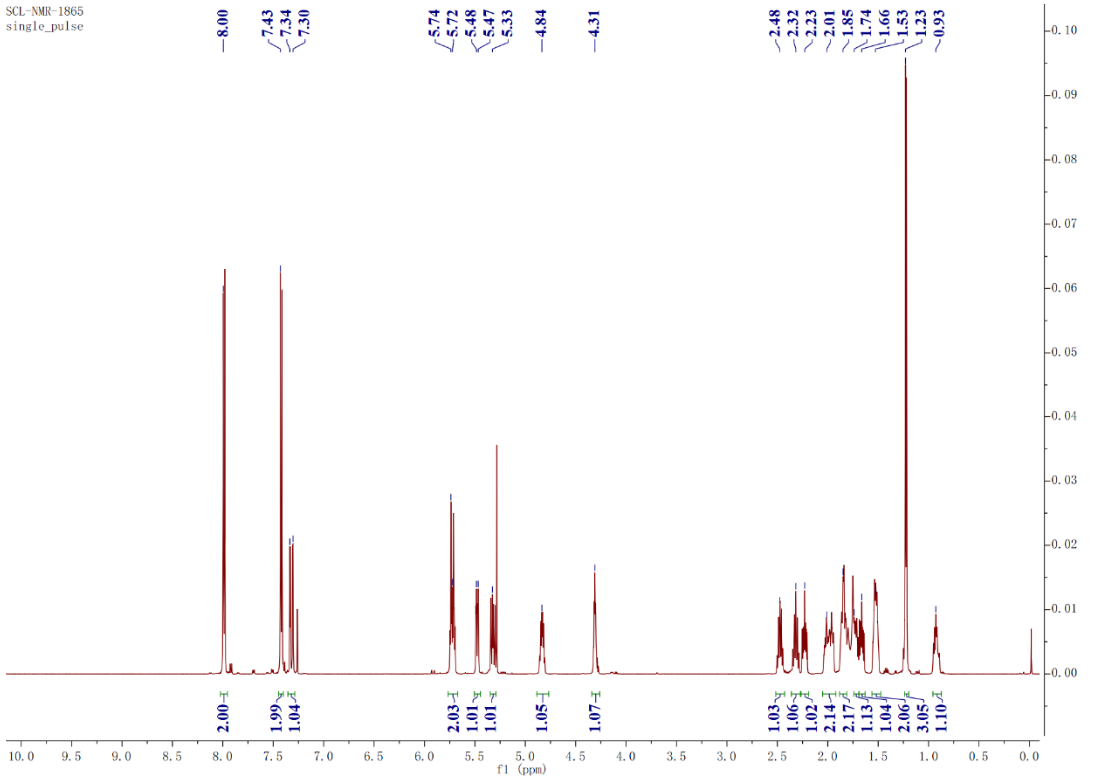

Figure S31. ^{1}H NMR (600 MHz, $CDCl_3$) spectrum of compound **11.**

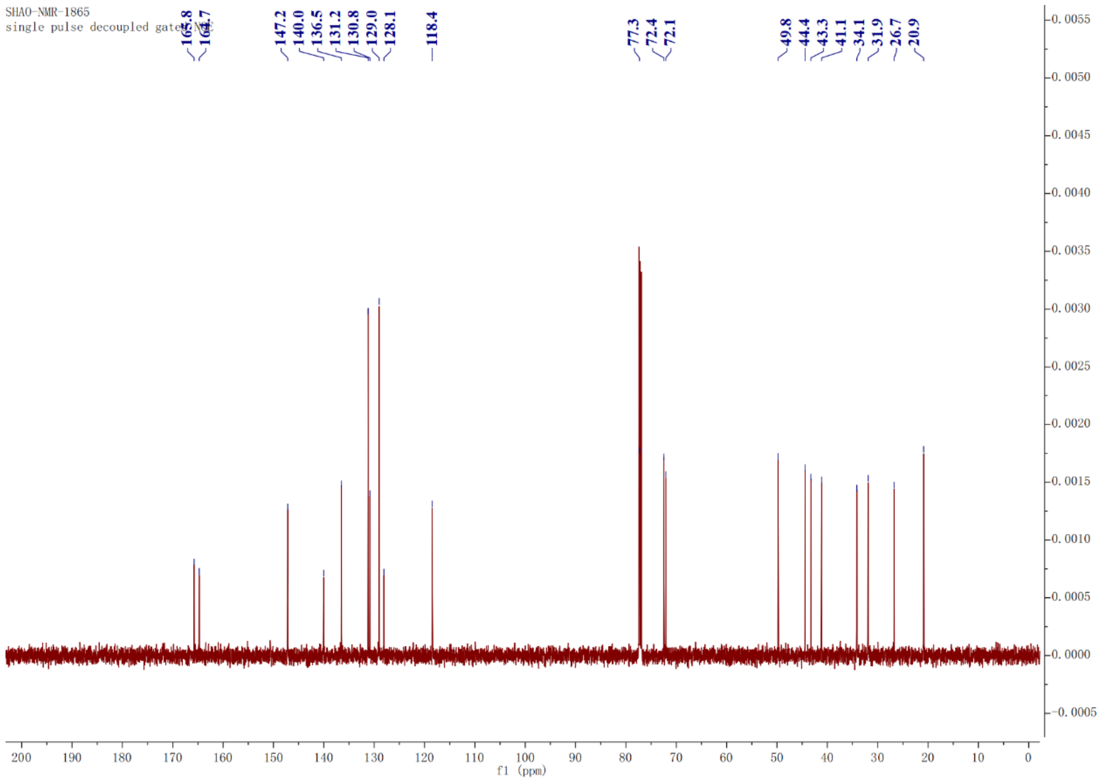

Figure S32. ^{13}C NMR (150 MHz, $CDCl_3$) spectrum of compound **11.**

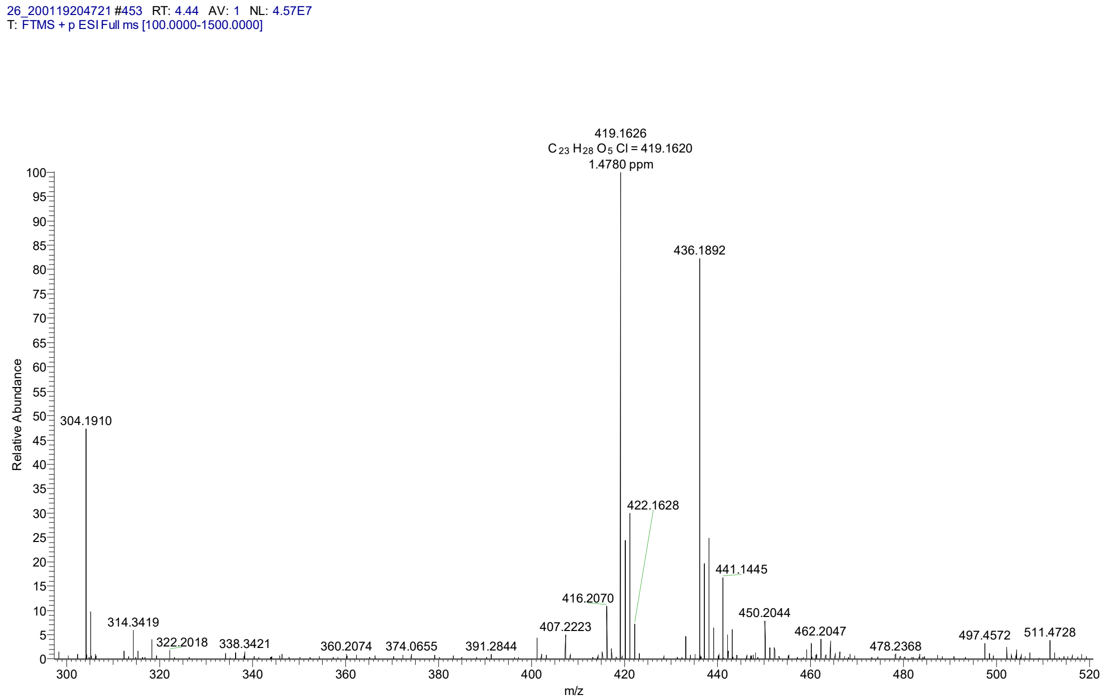

Figure S33. HRESIMS spectrum of compound **11**.

*Brefeldin A 4,7-O-di-(4)-chlorobenzoate (**12**)*

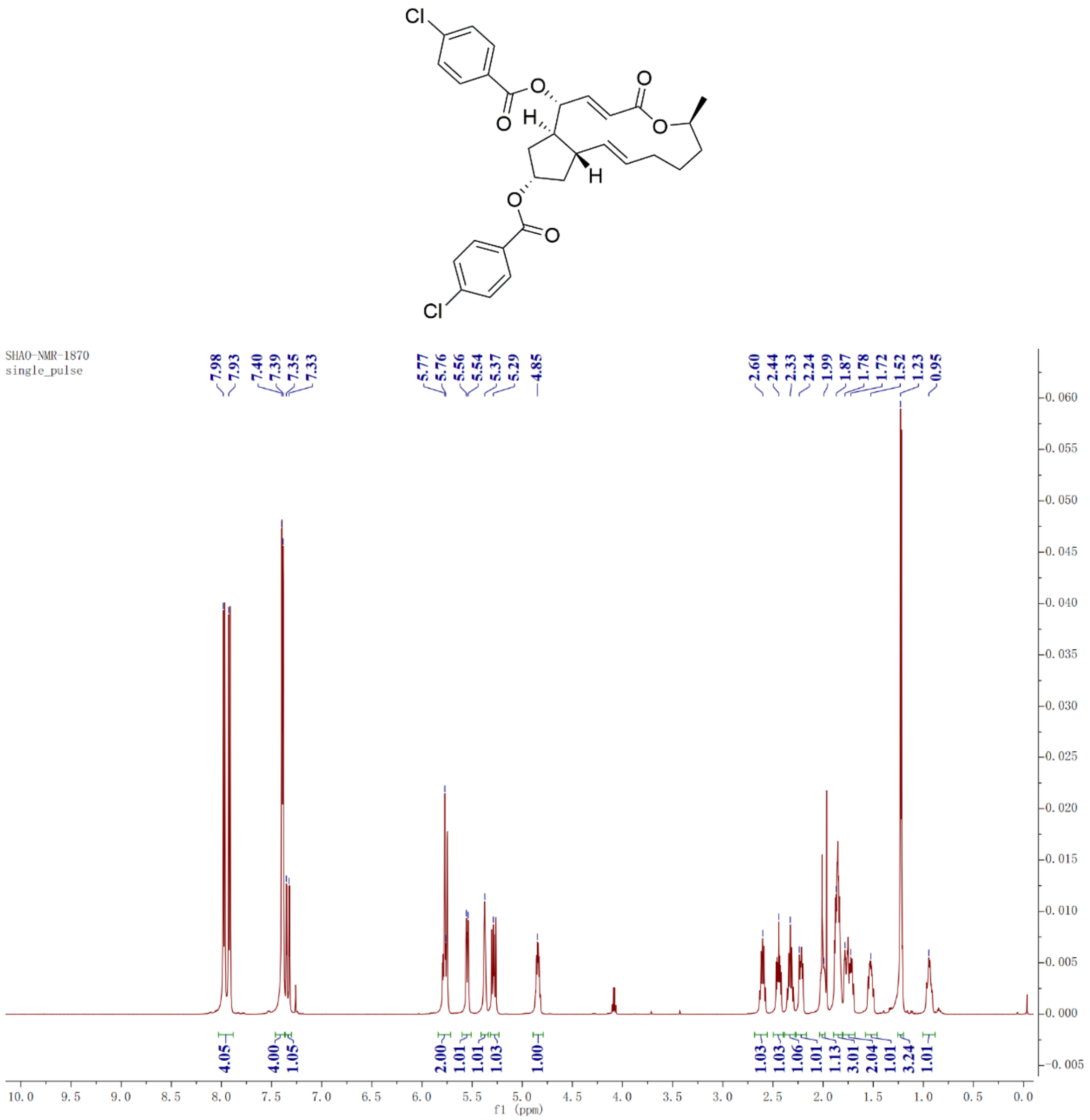

Figure S34. ^{1}H NMR (600 MHz, $CDCl_3$) spectrum of compound **12.**

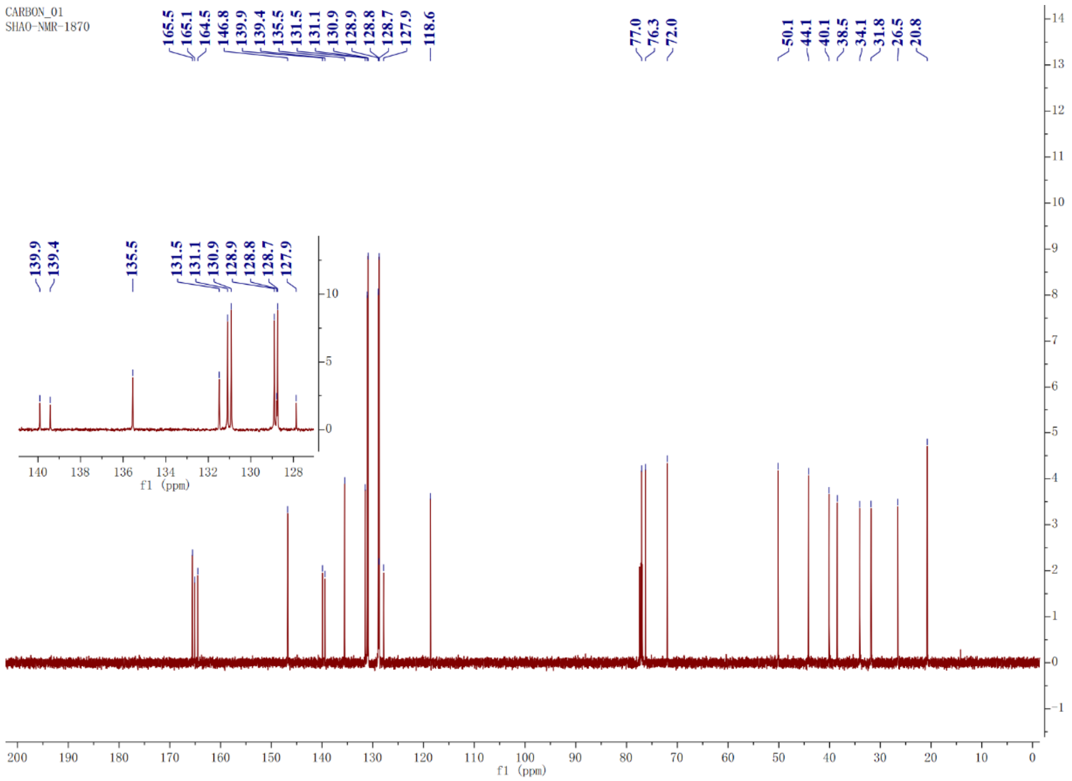

Figure S35. ^{13}C NMR (125 MHz, $CDCl_3$) spectrum of compound **12.**

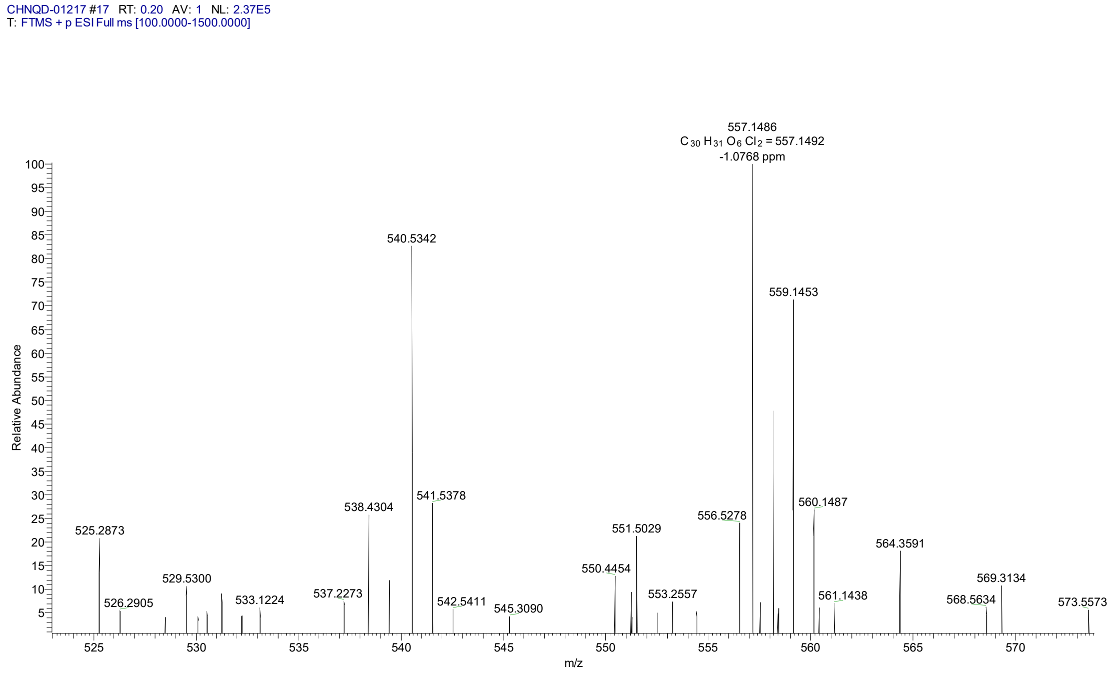

Figure S36. HRESIMS spectrum of compound **12**.

*Brefeldin A 7-O-2-chloro-4-fluorobenzoate (**13**)*

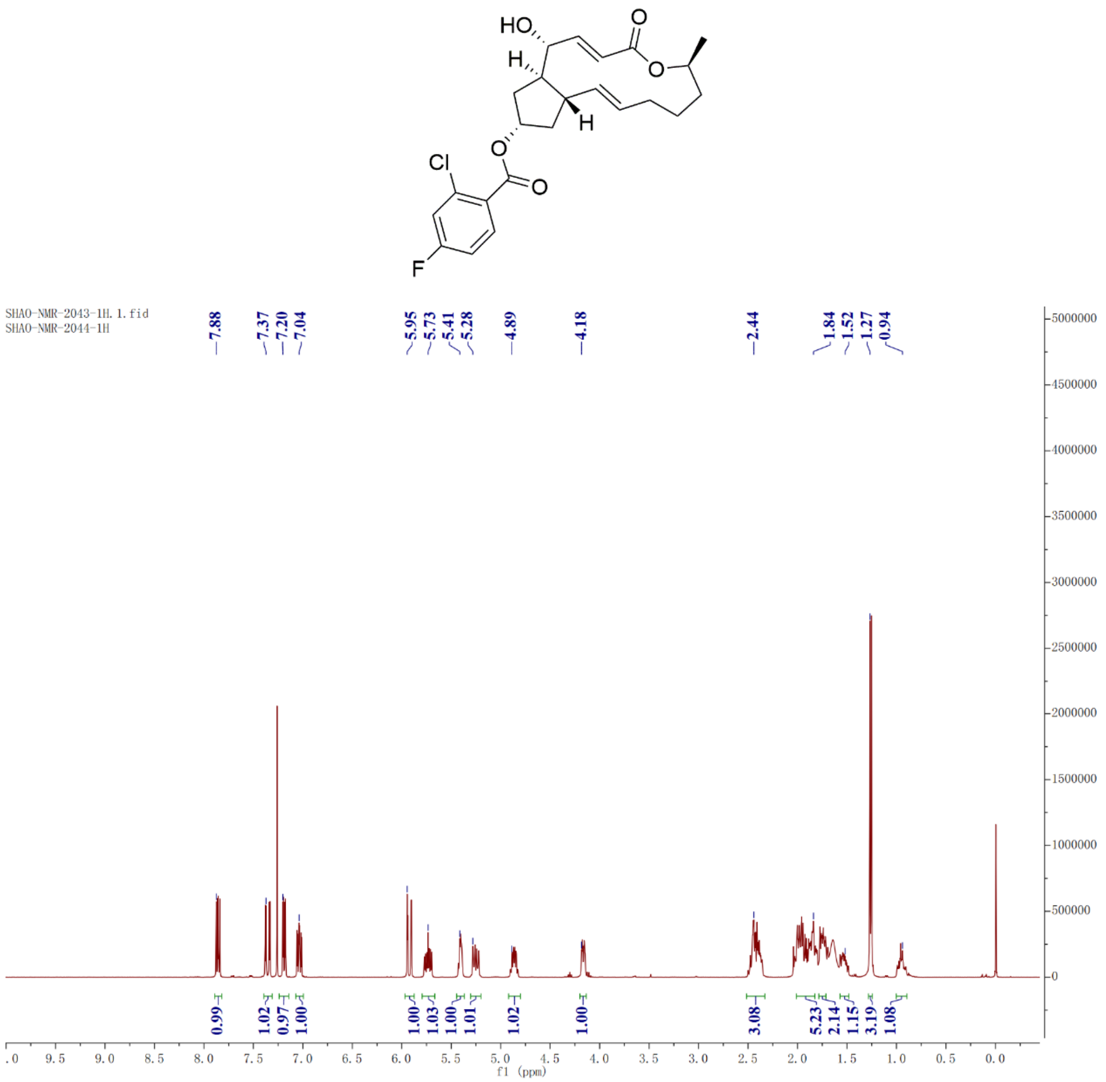

Figure S37. ^{1}H NMR (400 MHz, $CDCl_3$) spectrum of compound **13.**

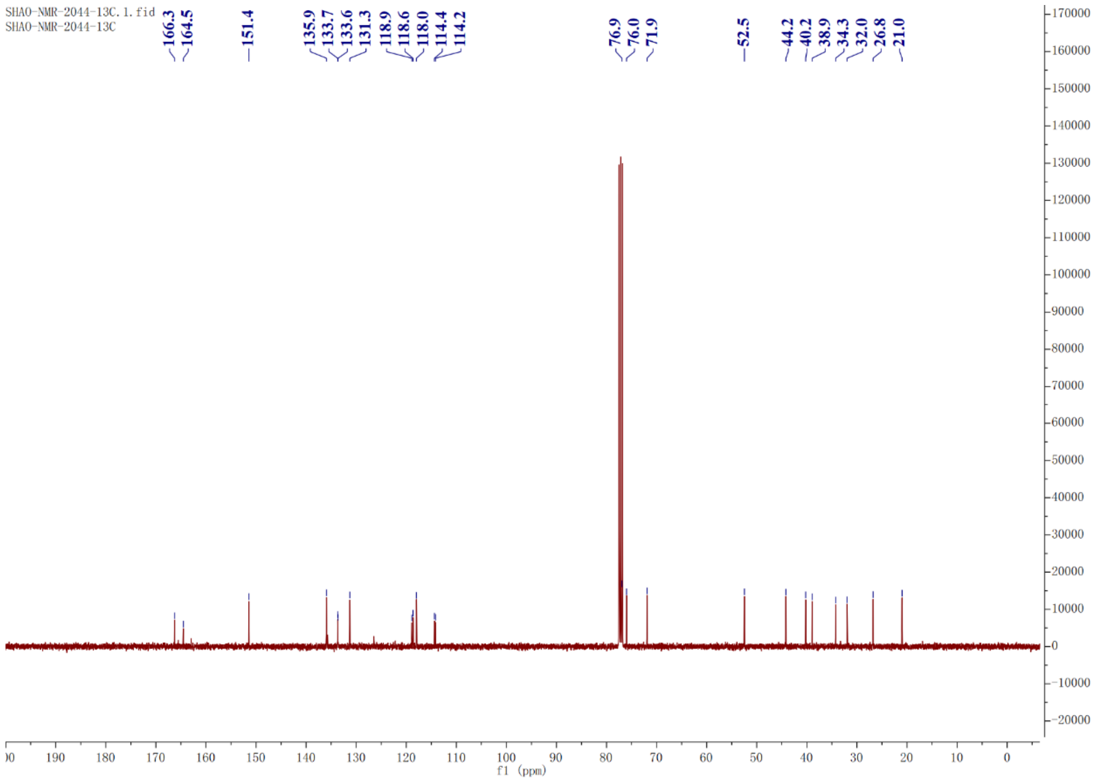

Figure S38. ^{13}C NMR (100 MHz, $CDCl_3$) spectrum of compound **13.**

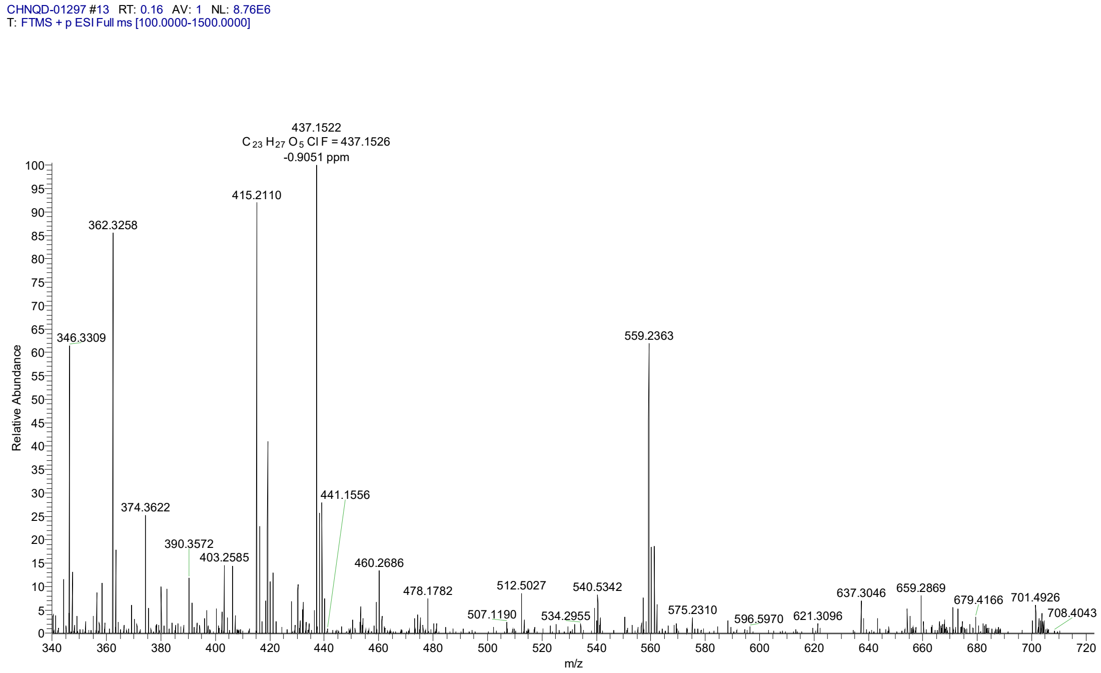

Figure S39. HRESIMS spectrum of compound **13**.

*Brefeldin A 4-O-2-chloro-4-fluorobenzoate (**14**)*

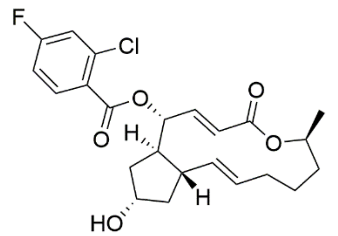

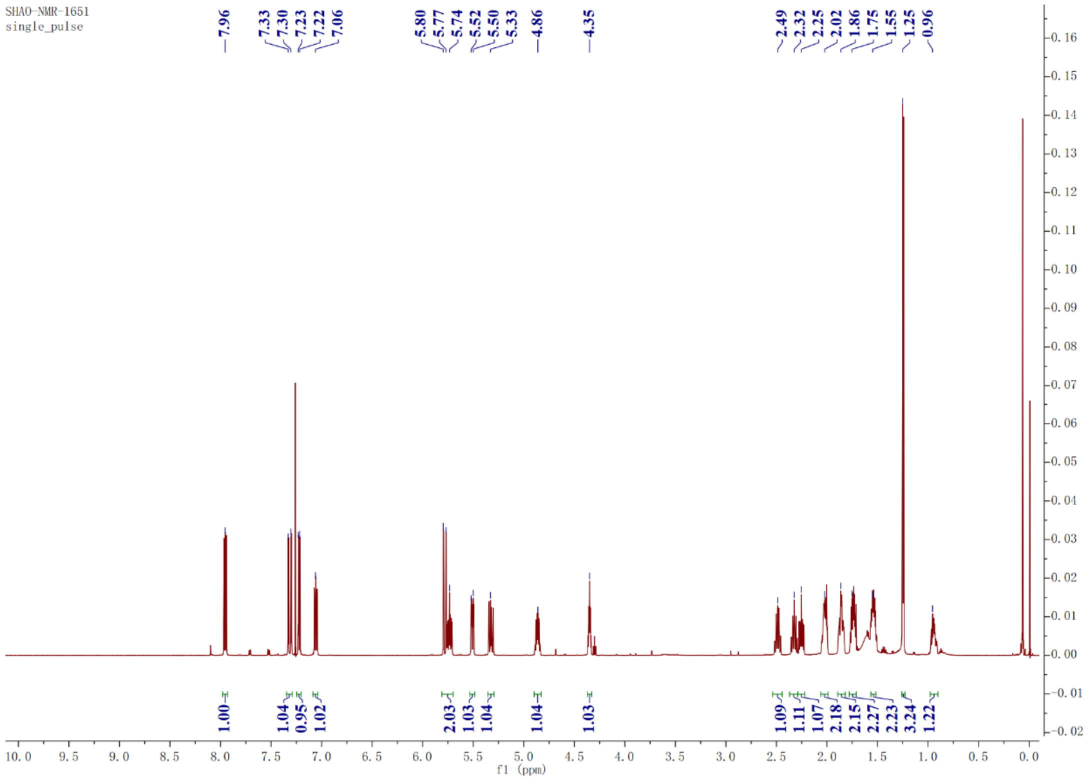

Figure S40. ^{1}H NMR (600 MHz, $CDCl_3$) spectrum of compound **14.**

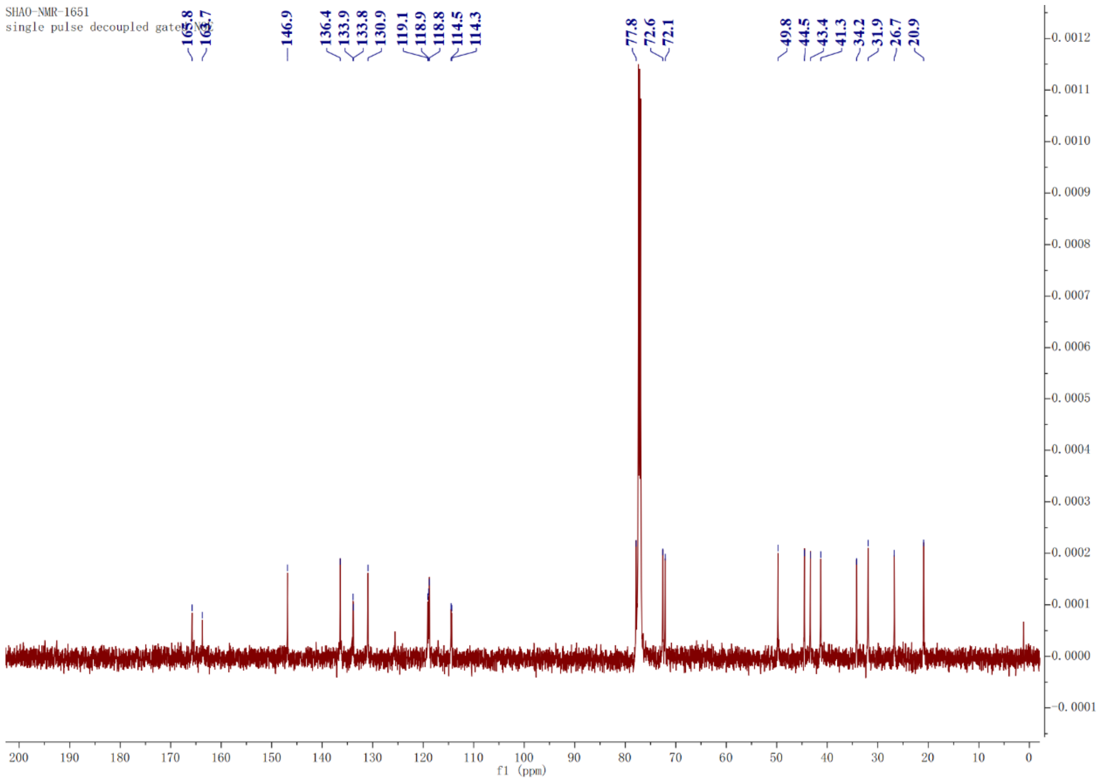

Figure S41. ^{13}C NMR (150 MHz, $CDCl_3$) spectrum of compound **14.**

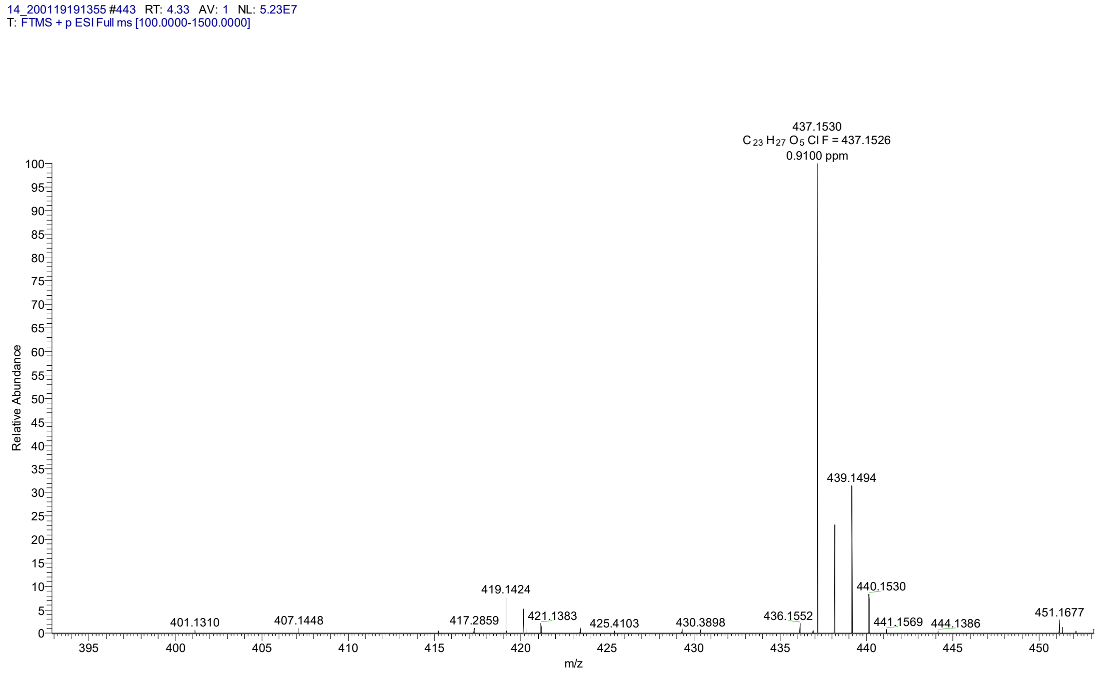

Figure S42. HRESIMS spectrum of compound **14**.

*Brefeldin A 4,7-O-di-2-chloro-4-fluorobenzoate (**15**)*

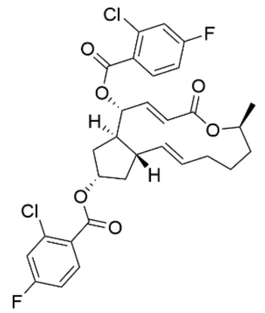

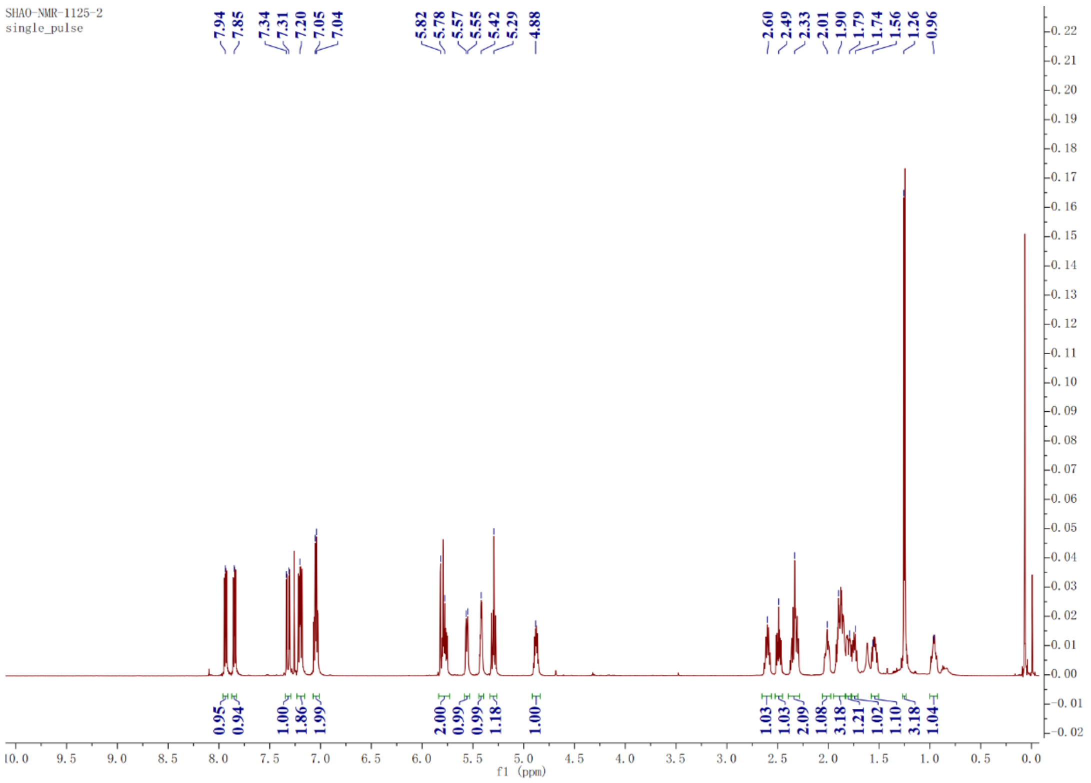

Figure S43. ^{1}H NMR (600 MHz, $CDCl_3$) spectrum of compound **15.**

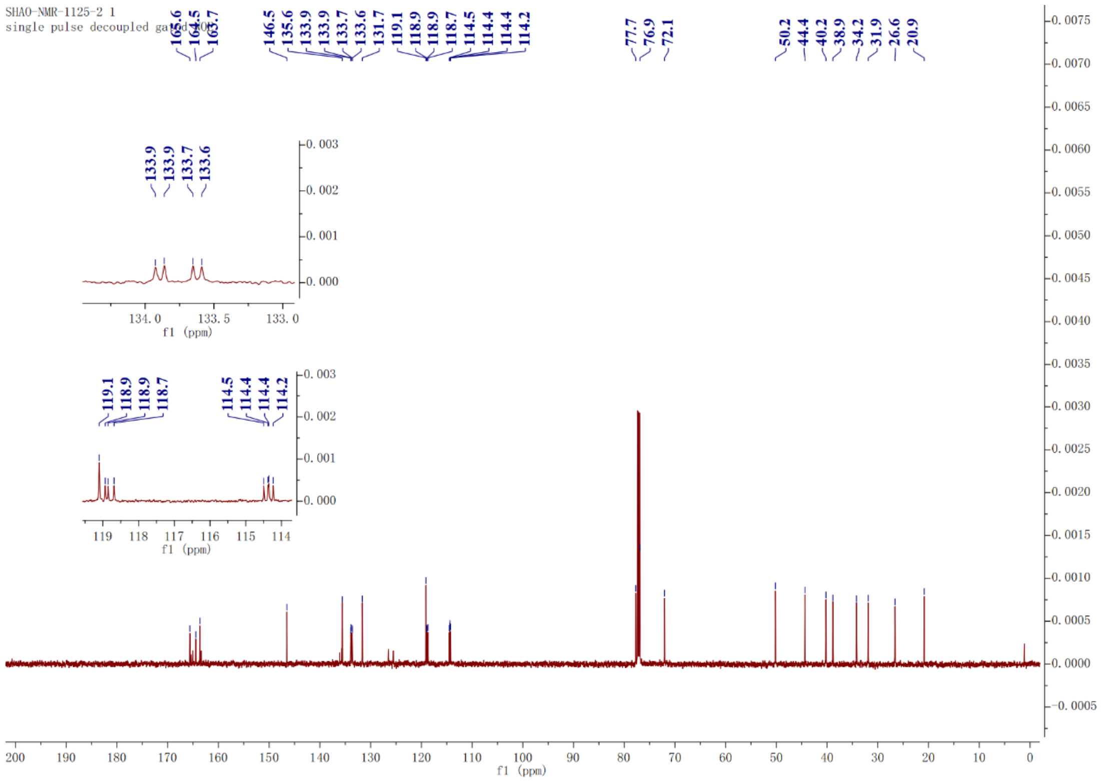

Figure S44. ^{13}C NMR (150 MHz, $CDCl_3$) spectrum of compound **15.**

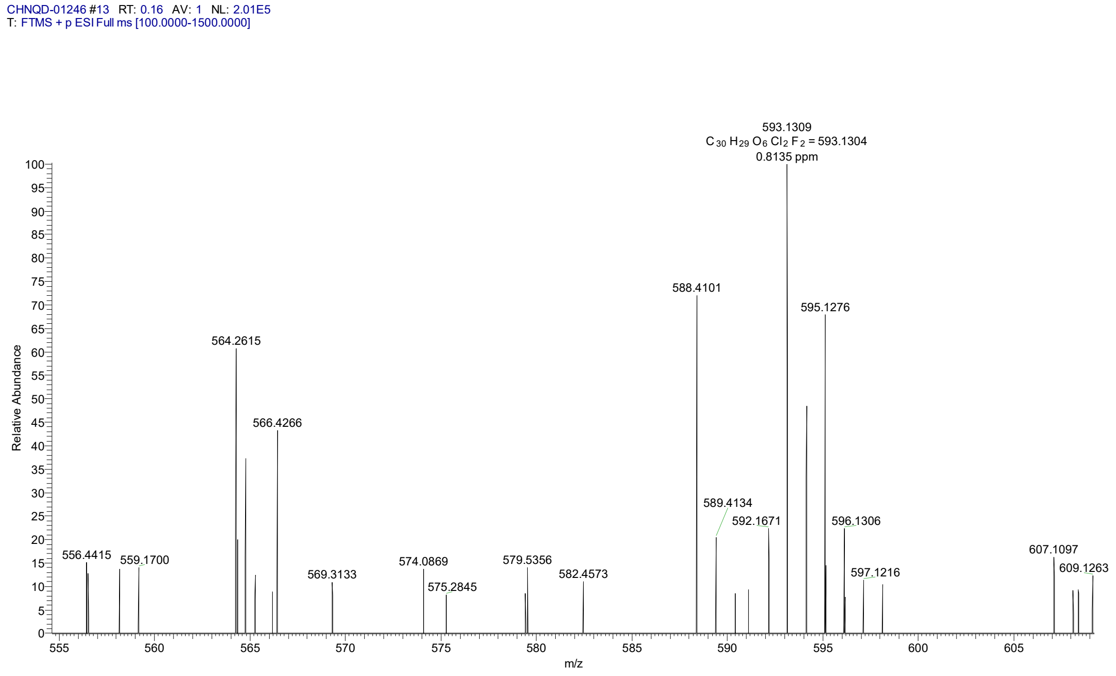

Figure S45. HRESIMS spectrum of compound **15**.

*Brefeldin A 7-O-(2,4,6)-trichlorobenzoate (**16**)*

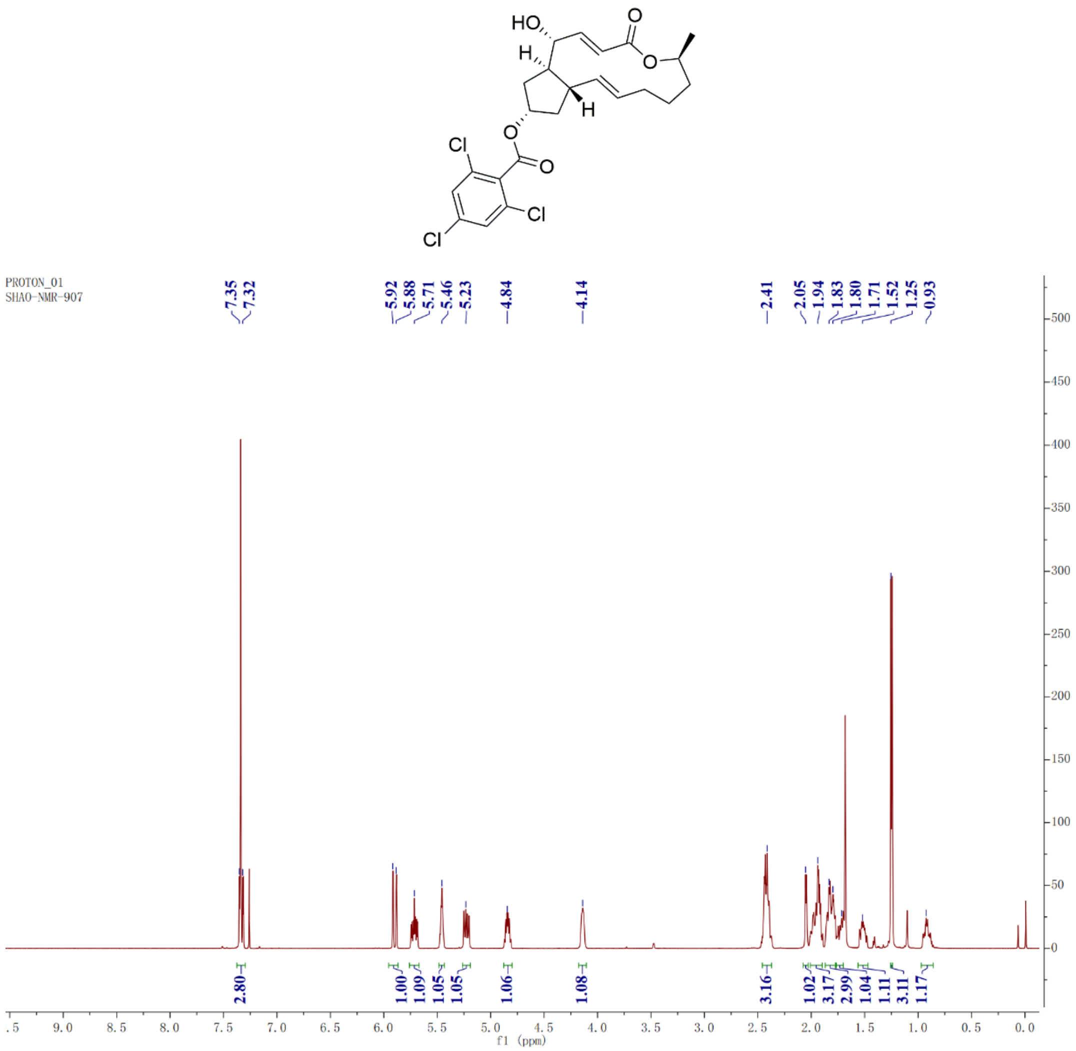

Figure S46. ^{1}H NMR (500 MHz, $CDCl_3$) spectrum of compound **16.**

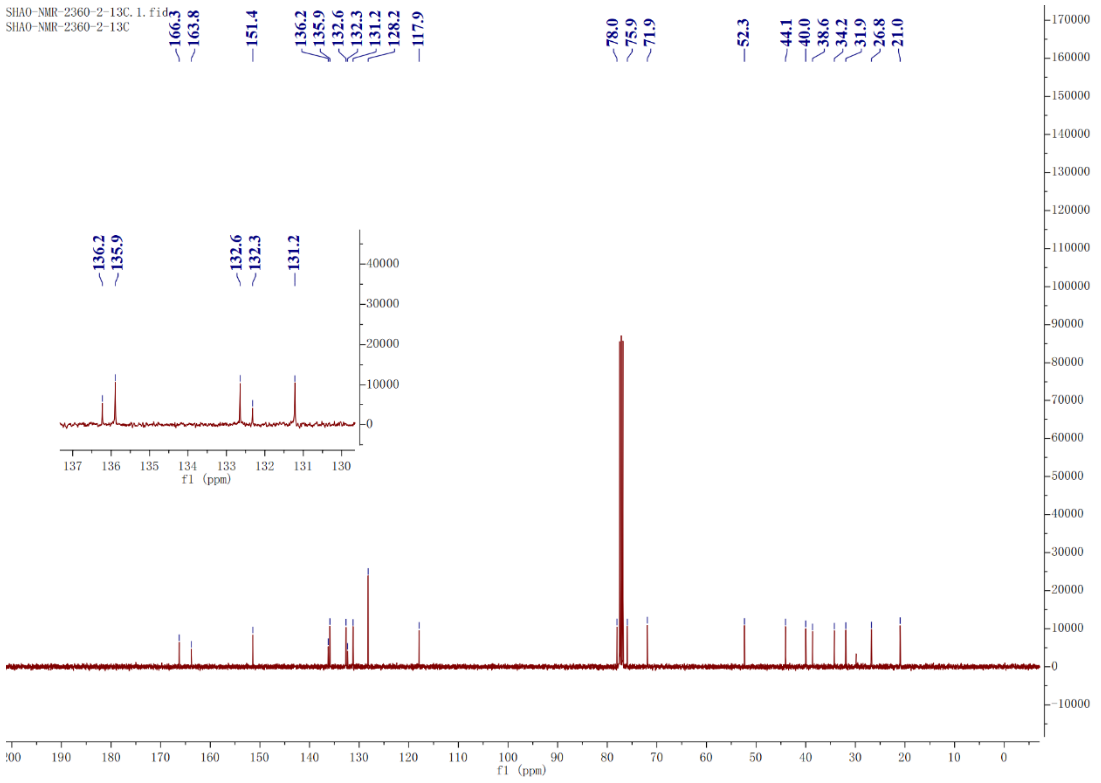

Figure S47. ^{13}C NMR (100 MHz, $CDCl_3$) spectrum of compound **16.**

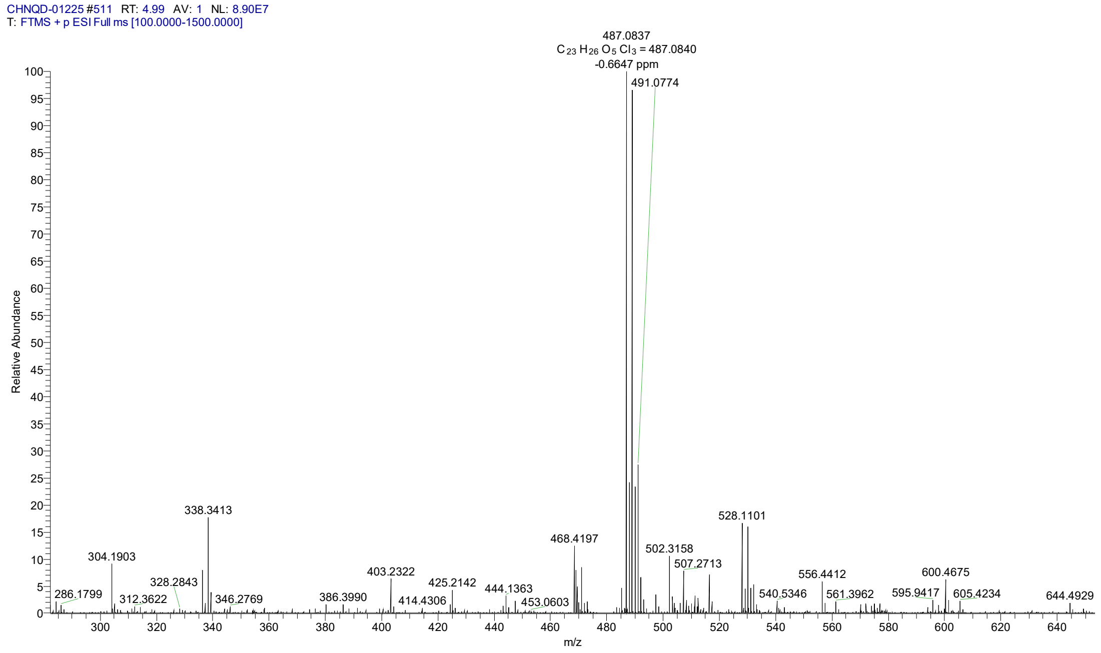

Figure S48. HRESIMS spectrum of compound **16**.
